# Polymerization Isomerism in Co-M (M = Cu, Ag, Au) Carbonyl Clusters: Synthesis, Structures and Computational Investigation

**DOI:** 10.3390/molecules26061529

**Published:** 2021-03-11

**Authors:** Cristiana Cesari, Beatrice Berti, Francesco Calcagno, Cristina Femoni, Marco Garavelli, Maria Carmela Iapalucci, Ivan Rivalta, Stefano Zacchini

**Affiliations:** Dipartimento di Chimica Industriale “Toso Montanari”, Università di Bologna, Viale Risorgimento 4, I-40136 Bologna, Italy; beatrice.berti6@unibo.it (B.B.); francesco.calcagno@studio.unibo.it (F.C.); cristina.femoni@unibo.it (C.F.); marco.garavelli@unibo.it (M.G.); maria.iapalucci@unibo.it (M.C.I.); i.rivalta@unibo.it (I.R.)

**Keywords:** metal cluster, carbonyl ligand, isomerism, molecular structure, DFT computations

## Abstract

The reaction of [Co(CO)_4_]^−^ (**1**) with M(I) compounds (M = Cu, Ag, Au) was reinvestigated unraveling an unprecedented case of polymerization isomerism. Thus, as previously reported, the trinuclear clusters [M{Co(CO)_4_}_2_]^−^ (M = Cu, **2**; Ag, **3**; Au, **4**) were obtained by reacting **1** with M(I) in a 2:1 molar ratio. Their molecular structures were corroborated by single-crystal X-ray diffraction (SC-XRD) on isomorphous [NEt_4_][M{Co(CO)_4_}_2_] salts. [NEt_4_](**3**)represented the first structural characterization of **3**. More interestingly, changing the crystallization conditions of solutions of **3**, the hexanuclear cluster [Ag_2_{Co(CO)_4_}_4_]^2−^ (**5**) was obtained in the solid state instead of **3**. Its molecular structure was determined by SC-XRD as Na_2_(**5**)·C_4_H_6_O_2_, [PPN]_2_(**5**)·C_5_H_12_ (PPN = N(PPh_3_)_2_]^+^), [NBu_4_]_2_(**5**) and [NMe_4_]_2_(**5**) salts. **5** may be viewed as a dimer of **3** and, thus, it represents a rare case of polymerization isomerism (that is, two compounds having the same elemental composition but different molecular weights) in cluster chemistry. The phenomenon was further studied in solution by IR and ESI-MS measurements and theoretically investigated by computational methods. Both experimental evidence and density functional theory (DFT) calculations clearly pointed out that the dimerization process occurs in the solid state only in the case of Ag, whereas Cu and Au related species exist only as monomers.

## 1. Introduction

Isomerism in molecular metal clusters is attracting considerable and renewed interest in view of its relevance to atomically precise metal nanoparticles, metal nanoclusters, ultrasmall metal nanoparticles and nanomaterials in general [1,2,3,4,5]. In analogy to organic and coordination chemistry, both stereoisomerism and structural isomerism have been observed in the field of gold nanoclusters and related molecular nanoclusters. These advancements have been possible owing the total structural determination of molecular nanoclusters and atomically precise metal nanoclusters by single-crystal X-ray diffraction (SC-XRD) [6,7,8,9,10]. Enantiomerism is the main type of stereoisomerism found up to now in molecular nanoclusters which, therefore, can be chiral [11,12]. Different types of structural isomerism have been revealed, including core (kernel) isomerism, staple (shell) isomerism and complex isomerism [13]. These show some analogies with chain, positional and functional isomerisms, which are well known in organic chemistry.

Isomerism is not limited to gold nanoclusters, but it is also well exemplified in other types of molecular clusters. In particular, molecular metal carbonyl clusters display a well-established chemistry and, in addition, they are good candidates from which to develop an organometallic approach to molecular metal nanoclusters [14]. Indeed, the first example of cluster core isomerism was reported several years ago for the [Pt_3_(μ-PPh_2_)_3_Ph(PPh_3_)_2_] organometallic cluster [15,16,17]. A similar core isomerism involving differences in the Pt-Pt contacts has been also reported for the Chini-type cluster [Pt_6_(CO)_10_(PPh_3_)_2_]^2−^ [18], as well as the solid state structures of homoleptic [Pt_3n_(CO)_6n_]^2−^ (*n* =5–8) Chini clusters [19,20]. Structural isomerism was then evidenced in Fe_2_Mo_2_(μ _3_-S)_2_(η^5^-C_5_H_5_)_2_(CO)_6_ (butterfly and planar isomers) [21] and [Au_3_{Fe(CO)_4_}_2_(PPh_3_)_2_]^−^ (linear and bent isomers) [22]. In addition, dynamic permutational isomerism was detected in the trigonal bypiramidal W_2_RhIr_2_(CO)_9_(η^5^-C_5_H_5_)_2_(η^5^-C_5_HMe_4_) cluster, resulting from competitive capping of either a W_2_Ir or WIr_2_ face of the cluster [23]. Surface isomerism is well represented in Ni and Co carbide carbonyl clusters decorated by Au(PPh_3_) fragments, such as Ni_6_C(CO)_9_(AuPPh_3_)_4_ [24], Co_5_C(CO)_11_(AuPPh_3_)_3_ [25] and Co_6_C(CO)_12_(AuPPh_3_)_4_ [26]. Four different isomers have been reported for [Ni_17_(C_2_)_2_(CO)_24_]^4−^, in which a Ni(CO) fragment may cap the [Ni_16_(C_2_)_2_(CO)_23_]^4−^ core of the cluster in four different modes [27]. Ligand isomerism has been also described in the case of [H_3_Ru_4_(CO)_12_]^−^, whose two isomers differ for the location of the three hydride ligands around the tetrahedral cage of the cluster [28]. Conversely, the two isomers of [Pt_9_(CO)_16_(R-dppp)]^2−^ (R-dppp = (*R*)-Ph_2_PCH(Me)(CH_2_PPh_2_) are due to two different orientations of the R-dppp ligand bonded to the surface of the cluster [29]. More recently, cluster core isomerism induced by acid-base reactions as well as crystal packing effects has been fully elucidated in the case of the [HCo_15_Pd_9_C_3_(CO)_38_]^2−^ molecular carbonyl nanocluster [30,31].

**Figure 1:**
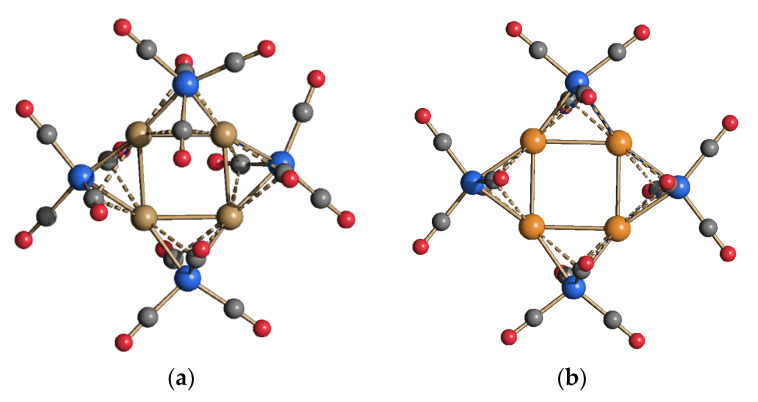

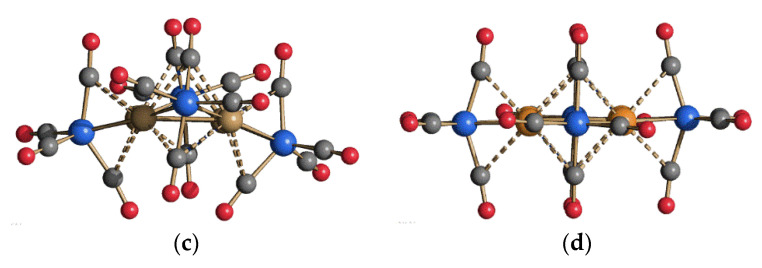
Two views of the molecular structures of (**a**,**c**) {CuCo(CO)_4_}_4_ [36] and (**b**,**d**) {AgCo(CO)_4_}_4_ (brown, Cu; orange, Ag; blue, Co; red, O; grey, C) [38].

**Figure 2:**
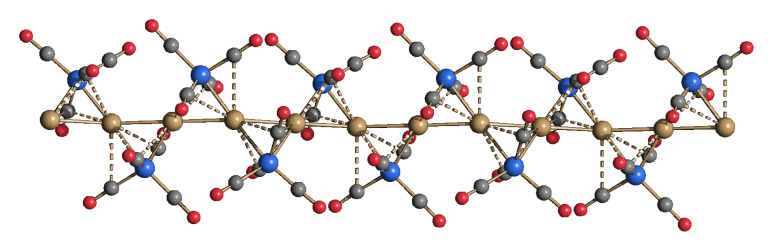
The molecular structure {CuCo(CO)_4_}_∞_ (brown, Cu; blue, Co; red, O; grey, C) [37].

Polymerization isomerism may be described as two compounds having the same elemental composition but different molecular weights [32,33]. This is a very rare phenomenon, both in coordination chemistry and molecular cluster chemistry. Probably, this term was applied for the first time to a metal carbonyl cluster in the case of the triangle and square polymerization isomers [{MFe(CO)_4_}_3_]^3−^ (M = Ag, Au) and [{MFe(CO)_4_}_4_]^4−^, which may be viewed as trimers and tetramers of [{MFe(CO)_4_}]^−^ [34]. Further examples of polymerization isomerism may be found in the literature concerning metal carbonyls, even if they were referred to differently. For instance, the Ru tetracarbonyl may exist as a Ru_3_(CO)_12_ triangular isomer (trimer), or as a [Ru(CO)_4_]_∞_ polymer [35]. A similar phenomenon has been observed in the case of the 1:1 adduct between Cu^+^ and [Co(CO)_4_]^−^ (**1**). Indeed, this may adopt the {CuCo(CO)_4_}_4_ square structure (tetramer) [36] or a {CuCo(CO)_4_}_∞_ polymeric structure (Figure 1 and Figure 2) [37]. In contrast, in the case of Ag, only the square tetramer {AgCo(CO)_4_}_4_ has been reported [38], whereas no Au related species has been described up to now. It must be remarked that {AgCo(CO)_4_}_4_ is perfectly planar, whereas {CuCo(CO)_4_}_4_ is not planar.

The M-Co(CO)_4_ (M = Cu, Ag, Au) system presents some further examples of structural isomerism in the case of the 1:2 adducts [M{Co(CO)_4_}_2_]^−^ (M = Cu, **2**; Ag, **3**; Au, **4**). In particular, three different isomers have been structurally characterized for **2** (see Results and Discussion) [39,40,41], whereas a single structure has been reported for **4** [42] and none for **3**.

In view of these interesting results and in order to better rationalize the M-Co(CO)_4_ (M = Cu, Ag, Au) system, herein we report a detailed study on the reactions between **1** and M(I) salts.

## 2. Results and Discussion

**Scheme 1:**
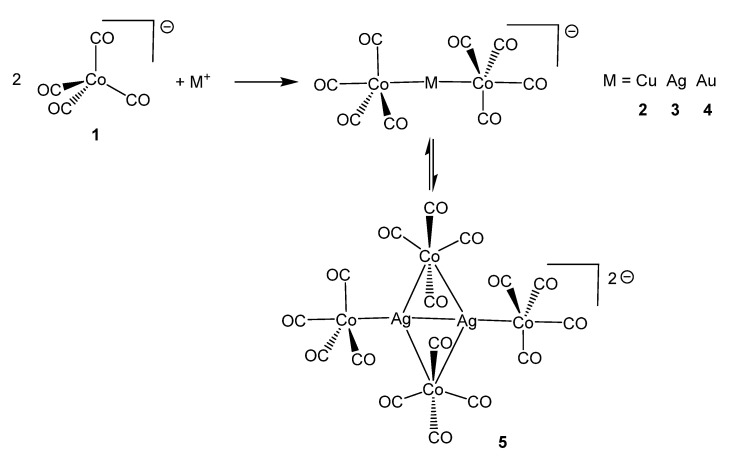
Syntheses of the complexes. M^+^ sources: [Cu(CH_3_CN)_4_][BF_4_], AgNO_3_, Au(Et_2_S)Cl.

The reaction of Na[Co(CO)_4_] (Na(**1**)) in CH_3_OH with M(I) salts and complexes ([Cu(CH_3_CN)_4_][BF_4_], AgNO_3_, Au(Et_2_S)Cl) in a 2:1 molar ratio afforded the [M{Co(CO)_4_}_2_]^−^ anions (M = Cu, **2**; Ag, **3**; Au, **4**) (Scheme 1) as indicated by their typical ν_CO_ bands in the IR spectra (Figures S1–S5 in the Supporting Information) [39,40,41,42,43]. At the end of the reaction, **2**–**4** have been precipitated as [NEt_4_]^+^ salts by addition of a saturated solution of [NEt_4_]Br in H_2_O. Crystallization from CH_2_Cl_2_/n-pentane afforded crystals of [NEt_4_](**2**), [NEt_4_](**3**) and [NEt_4_](**4**) suitable for SC-XRD, that confirmed the structures of the **2**–**4** monoanions. It is noteworthy that [NEt_4_](**3**) represents the first structural characterization of **3**.

**Figure 3:**
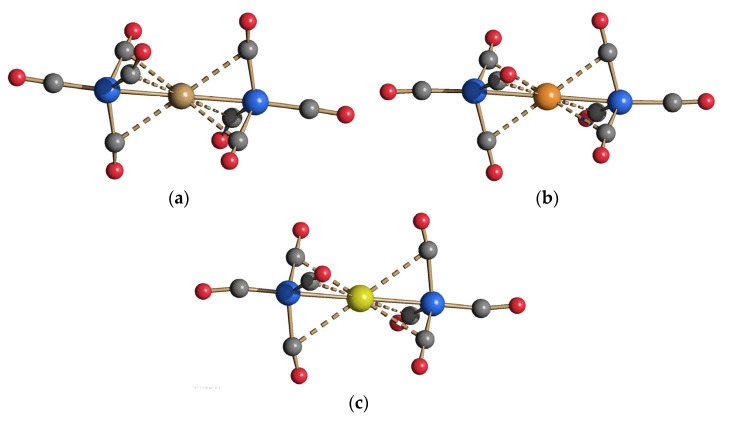
Molecular structures of (**a**) [Cu{Co(CO)_4_}_2_]^−^ (**2**), (**b**) [Ag{Co(CO)_4_}_2_]^−^ (**3**), and (**c**) [Au{Co(CO)_4_}_2_]^−^ (**4**). Sub Van der Waals M···C(O) contacts are represented as fragmented lines (brown, Cu; orange, Ag; yellow, Au; blue, Co; red, O; grey, C). All the anions adopt the TBP-TBP staggered structure.

**Figure 4:**
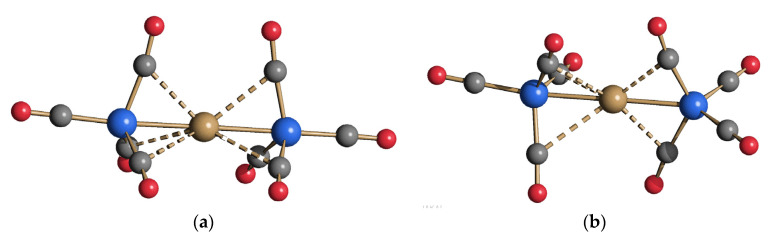
Molecular structures of **2** as found in (**a**) [Cu(dmpe)_2_](**2**) (eclipsed conformation; TBP-TBP eclipsed isomer) and (**b**) [PPN](**2**) (TBP-Td isomer). Sub Van der Waals M**···**C(O) contacts are represented as fragmented lines (brown, Cu; blue, Co; red, O; grey, C) [40].

The crystals of [NEt_4_](**2**), [NEt_4_](**3**) and [NEt_4_](**4**) are isomorphous and consist of an ionic packing of [NEt_4_]^+^ cations and [M{Co(CO)_4_}_2_]^−^ anions (Figure 3 and Table 1). The structures of **2** and **4** were previously reported as different salts [39,40,41,42], whereas the one herein reported represents the first structural determination of **3**, even if its synthesis was previously reported [43]. The M(I) ion of **2**–**4** displays a linear coordination and the two Co-centers adopt both a trigonal bipyramidal (TBP) geometry as previously found in [Cu(IMes)_2_](**2**) (IMes = C_3_N_2_H_2_(C_6_H_2_Me_3_)_2_ [39], [Cu(dmpe)_2_](**2**) (dmpe = Me_2_PCH_2_CH_2_PMe_2_ [40], [Cu(P{OMe)_3_}_4_](**2**) [41] and [PPN](**4**) (PPN = N(PPh_3_)_2_]^+^) [42]. The two Co(CO)_4_ groups of **2**–**4** adopt a staggered conformation in all the salts, including those described in this paper. An exception is represented by [Cu(dmpe)_2_](**2**) [40], whose unit cell contains two independent anions, both containing two TBP-Co(CO)_4_ groups, but one anion adopts a staggered conformation and the other an eclipsed conformation (Figure 4). A similar phenomenon has been observed for Hg{Co(CO)_4_}_2_, in which the two TBP-Co(CO)_4_ groups may adopt a staggered or eclipsed conformation, and both have been characterized by SC-XRD [44].

**Figure 5:**
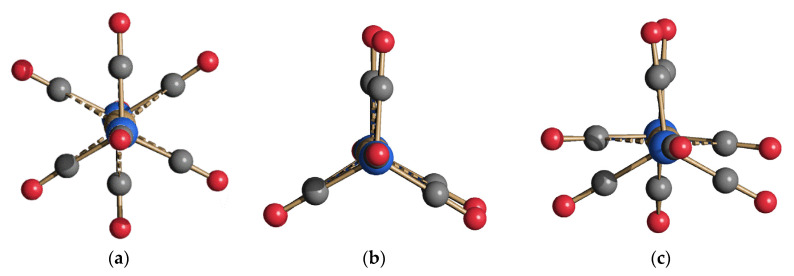
The three isomers of **2**: (**a**) TBP-TBP staggered; (**b**) TBP-TBP eclipsed; (**c**) TBP-Td. Sub Van der Waals M**···**C(O) contacts are represented as fragmented lines (brown, Cu; blue, Co; red, O; grey, C) [39,40,41].

A further isomer of **2** was reported as [PPN](**2**) salt [41], where a Co center is TBP and the second one displays a tetrahedral coordination of the four CO ligands, with Cu capping one edge of the Co(CO)_4_ tetrahedron (Figure 4). Thus, three different isomers of **2** have been reported up to now: (a) TBP-TBP staggered; (b) TBP-TBP eclipsed; (c) TBP-Td (Figure 5). The Cu-Co distances in the structures reported are comprised in a very narrow range [2.326–2.411 Å] and are comparable in all the isomers. Notably, DFT computations indicate that the TBP-TBP staggered (a) is the most stable conformer of the TBP-TBP isomer in the gas phase (see Figure S0 in the Supporting Information) and in the presence of a solvent dielectric. The geometry optimizations of the (a), (b) and (c) isomers of **2**, indeed, all converge into the TBP-TBP staggered (a) isomer. However, if the [PPN]^+^ counterion is placed close by the Cu complex, a local minimum with TBP-Td geometry can be found (see Figure S0 in the Supporting Information), in agreement with the experimental evidence for [PPN](**2**) salt [41].

The reactions of [PPN](**1**) with M(I) salts resulted in species showing ν_CO_ bands in the IR spectra similar to **2**–**4**. Indeed, crystals of [PPN](**2**) and [PPN](**4**) suitable for SC-XRD were obtained and these displayed the same cell parameters and crystal structures previously reported in the literature for the **2** and **4** monoanions with the same cation [41,42]. Surprisingly, in the case of M = Ag, crystals of [PPN]_2_[Ag_2_{Co(CO)_4_}_4_]·C_5_H_12_ were obtained, which contained the [Ag_2_{Co(CO)_4_}_4_]^2−^ dianion (**5**). We may view **5** as a dimer of **3**, and all the experimental evidence (see below) points out that **5** is formed upon crystallization. In particular, the ESI-MS analyses of both crystals of **3** and **5** (see below) indicate that only the monomer **3** is detected in solution, regardless of the species present in the solid state. A potential equilibrium between **3** and **5** has been theoretically investigated by DFT computations (see Scheme S1 in the Supporting Information), showing that **3** is slightly more stable than **5**. Nonetheless, there is no experimental evidence of the presence of **5** in solution, which is, therefore, formed in the solid state upon crystallization. This represents a further case of polymerization isomerism, since **3** and **5** have the same elemental compositions but different molecular weights.

In order to shed light on this point, the crystallization of the other salts of the product of the reaction of **1** and Ag^+^ in a 2:1 molar ratio was attempted, following similar procedures to that described above (see Experimental for details). This resulted in the structural characterization by SC-XRD of four new salts, that is Na_2_(**5**)·C_4_H_6_O_2_, [PPN]_2_(**5**)·C_5_H_12_, [NBu_4_]_2_(**5**) and [NMe_4_]_2_(**5**). All of them contain the dimeric dianion **5**. Thus, it is possible to assume that depending on the crystallization conditions, either salts of **3** and **5** can be obtained. Conversely, in the case of Cu and Au, only the monomers **2** and **4** have been observed and structurally characterized up to now. DFT calculations (see Scheme S1 in the Supporting Information) suggest that these observations rely on a different thermodynamic profile of the Ag system compared to Cu and Au ones. Thus, the monomer and dimer display very similar energies for Ag, whereas the monomer is largely favored in the case of Cu and Au.

The IR spectra obtained upon dissolving [NEt_4_](**2**), [NEt_4_](**3**), [NEt_4_](**4**) and [NMe_4_]_2_(**5**) crystals in CH_2_Cl_2_ solutions are very similar (Figures S1–S5 in the Supporting Information). In particular, they show two intense ν_CO_ bands at 2026(s) and 1945(vs) cm^−1^ (**2**); 2026(s) and 1938(vs) cm^−1^ (**3**); 2025(s) and 1957(vs) cm^−1^ (**4**); 2027(s) and 1938(vs) cm^−1^ (**5**). The bands at 1938–1957 cm^−1^ feature a significant asymmetric broadening towards lower frequencies while the narrow bands at 2025–2027 cm^−1^ show a shoulder at higher frequencies around 2040 cm^−1^. From the similarities in the experimental IR spectra, it can be concluded that the monomers **2**–**4** are present in solution, since **2** and **4** also exist only as monomers in the solid state. The spectra of **3** and **5** in solution are almost identical, indicating that mainly (or only) one species is actually present in solution. Indeed, it is likely that in the case of Ag, **3** is the main (or almost the only) species present in solution, whereas **5** is formed only upon crystallization. This point has been further corroborated by ESI-MS analyses.

DFT computations provided useful insights into the experimentally observed lineshapes of IR spectra. In particular, we monitored various effects that can shape the IR spectra of complex **3** (see Figures S6 and S7 in the Supporting Information). The simulated IR spectrum of **3** in the gas phase (see Figure S6) features the same two main bands observed experimentally but with an underestimated relative frequency gap (ca. 60 vs. 80 cm^−1^). The addition of an implicit solvent model, which takes into account the effect of the solvent dielectric, results into a larger frequency gap, with the modes at ca. 1938–1957 cm^−1^ being red-shifted and split so that the IR band is broadened. By including the explicit effect of the local interactions between **3** and the dichloromethane solvent (see Figure S7), the DFT simulated spectrum features a broadening of the band at ca. 1938–1957 cm^−1^, in line with experimental spectral lineshape.

The IR spectra of [NEt_4_](**3**) and [NMe_4_]_2_(**5**) registered in the solid state by ATR mode are sensibly shifted to lower frequencies compared to those recorded in solution (Figures S8–S11 in the Supporting Information). In order to obtain information on the red-shift observed in solid state ATR spectra with respect to IR spectra in solution, we performed DFT simulations of the IR spectrum of **3** in the presence of an explicit molecule of the [NEt_4_]^+^ counterion (see Figure S12 in the Supporting Information), assuming a tight ion-pair conformation as observed in the solid state structure. The tight ion-pair spectrum shows a significant broadening of the main band of **3** in solution (at ca. 1938–1957 cm^−1^), with a sizeable red-shift of the stretching modes for the CO groups point towards the [NEt_4_]^+^ counterion, which agrees well with the red-shifted band in the ATR spectrum. By including a nearby counterion molecule in the model, also the 2040 cm^−1^ shoulder of the experimental narrow band at 2025–2027 cm^−1^ is recovered. These results suggest that the experimental lineshape of **3** is dominated by the distortion of symmetry induced by local interactions with counterions (or, eventually, close by solvent molecules).

In order to further investigate the nature of the species present in solution, ESI-MS studies on CH_3_OH solutions of **2**–**5** have been carried out (Figures S13–S19 and Tables S1–S3 in the Supporting Information). All anions have been studied as [PPN]^+^ salts, in order to avoid ion pairing in the gas phase. As expected, only the monomers [M{Co(CO)_4_}_2_]^−^ have been detected in the case of the Cu and Au salts [PPN](**2**) and [PPN](**4**). Indeed, the ESI-MS spectrum (ES–) of [PPN](**4**) displays a very intense peak at *m*/*z* 539 corresponding to the molecular ion [Au{Co(CO)_4_}_2_]^−^. Its monoanionic charge is confirmed by the presence of a minor peak at m/z 511, that corresponds to the loss of a single CO ligand. Further, the simulated isotopic pattern of all the peaks agrees with the monomeric nature of **4** in solution. Similarly, an intense peal at *m*/*z* 405 corresponding to [Cu{Co(CO)_4_}_2_]^−^ has been detected in the ESI-MS spectrum (ES–) of [PPN](**2**).

Surprisingly, the ESI-MS spectrum (ES–) of [PPN]_2_(**5**) (Figures S17–S19 in the Supporting Information) also indicates that only the monomer [Ag{Co(CO)_4_}_2_]^−^ (**3**) is present in solution, despite the fact that the dimer [Ag_2_{Co(CO)_4_}_4_]^2−^ (**5**) is present within the crystals. Indeed, the very strong peak of the molecular ion at *m*/*z* 449 shows the typical isotopic pattern of an ion that contains a single Ag atom. Comparison of the experimental peak with the calculated ones for **3** and **5** (Figure S18 in the Supporting Information), completely rules out the presence of even traces of the dimer in solution. This point is further corroborated by the presence of a peak at *m*/*z* 421 attributable to the loss of one CO ligand (28 amu) from the monoanionic molecular ion. It must be concluded that the dimer **5** is formed during crystallization.

It must be remarked that similar results have been obtained both by analyzing the crystals of compounds **2**–**5** by ESI-MS as well as by performing the ESI-MS analyses on the solutions obtained from the reactions of **1** and M^+^ salts before crystallization. Thus, it may be concluded that the monomers [M{Co(CO)_4_}_2_]^−^ (M = Cu, **2**; Ag, **3**; Au, **4**) are the only species present in solution (at least to the limit of detection of the employed analytical techniques), whereas the dimer **5** is observed only in the solid state. Its formation might be due to packing effects. We cannot rule out the presence in solution of an equilibrium between **3** and **5**, where **3** is the prevalent species and **5** is present in a very small amount that escapes any available analytical techniques. Nonetheless, there is no clear experimental evidence for the presence of **5** in solution at the moment.

**Figure 6:**
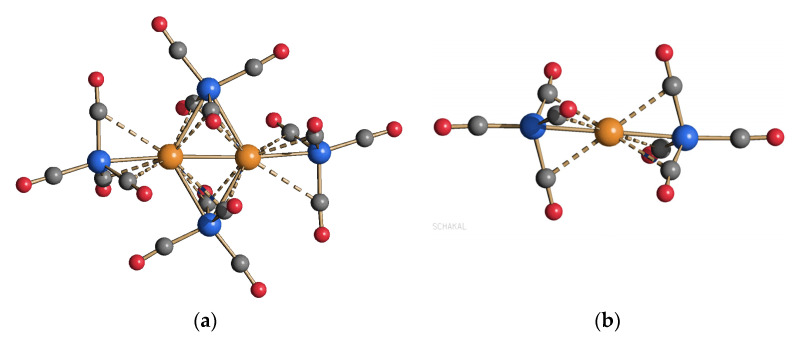
Molecular structure of (**a**) the dimer [Ag_2_{Co(CO)_4_}_4_]^2−^ (**5**) compared to (**b**) the monomer **3**. Sub Van der Waals Ag**···**C(O) contacts are represented as fragmented lines (orange, Ag; blue, Co; red, O; grey, C).

The structure of the new anion **5** (Figure 6) has been determined as four different salts, that is Na_2_(**5**)·C_4_H_6_O_2_, [PPN]_2_(**5**)·C_5_H_12_, [NBu_4_]_2_(**5**) and [NMe_4_]_2_(**5**), displaying very similar geometries and bonding parameters. We may view **5** as the dimer of **3** and, thus, **3/5** represents a further example of polymerization (monomer/dimer) isomerism in carbonyl clusters. The dimeric structure of **5** is also unprecedented for Cu and Au. It is composed by an Ag_2_ unit bonded to two terminal (Co_t_) and two edge bridging (Co_b_) Co(CO)_4_ units. The Ag-Ag contact is slightly different in the four salts (2.8425(3) Å for Na_2_(**5**)·C_4_H_6_O_2_, 2.8775(7) Å for [PPN]_2_(**5**)·C_5_H_12_, 2.91970(17) Å for [NBu_4_]_2_(**5**), 2.916(4) and 2.923(4) Å for [NMe_4_]_2_(**5**)) and is indicative of an argentophilic interaction as found in other Ag clusters supported by organometallic carbonyl fragments [45,46,47]. As expected, the Co_t_-Ag contacts [2.6537(3), 2.6692(7), 2.67936(18), 2.669(4) and 2.679(4) Å for the four salts] are shorter than Co_b_-Ag [2.8576(3), 2.8094(10), 2.75393(19)–2.8475(2) and 2.787(4)–2.805(4) Å]. Moreover, the Co_t_-Ag distance of **5** is longer than in **3** [2.4392(17)–2.4450(17) Å], in view of the fact that Ag displays coordination number two in **3**, and three (four considering also the Ag-Ag contact) in **5**. Indeed, the Ag-Co contact [2.75 Å] in the mononuclear complex Co(CO)_4_{AgAs_3_(CH_3_)_5_(C_6_H_4_)_2_}, which contains an Ag center strongly bonded to three As atom (Ag coordination number 4), is even longer than in **5** [48].

**Figure 7:**
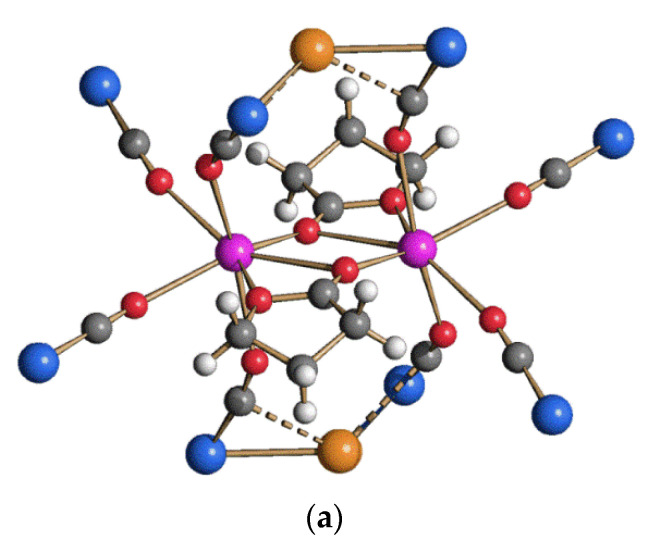

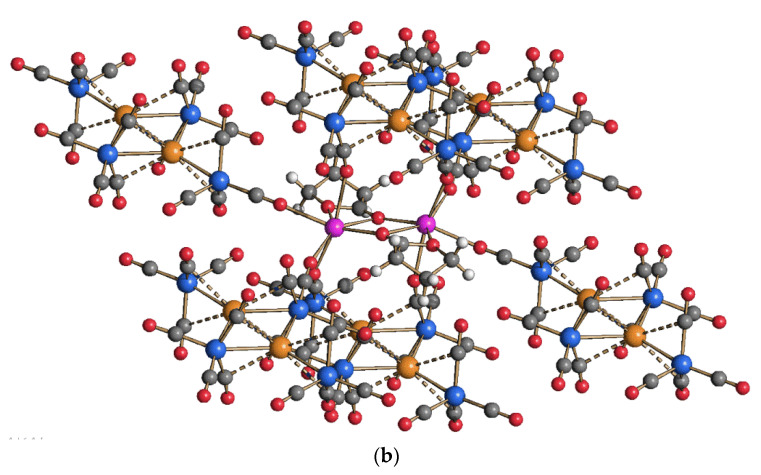
View of (**a**) the Na^+^ coordination and (**b**) the isocarbonyl linkages present in the crystal packing of Na_2_(**5**)·C_4_H_6_O_2_. Sub Van der Waals Ag**···**C(O) contacts are represented as fragmented lines (orange, Ag; blue, Co; red, O; grey, C; white, H; purple, Na).

The crystal packing of Na_2_(**5**)·C_4_H_6_O_2_ contains an interesting network of isocarbonyl linkages involving the Na^+^ ions (Figure 7). Indeed, each Na^+^ is coordinated to the O-atoms of four CO ligands of four different **5** anions. The overall coordination number of each Na^+^ ion is seven, being coordinated to the endo-cyclic O-atom of one cocrystallized γ-butyrolactone C_4_H_6_O_2_, the exo-cyclic O-atom of the same C_4_H_6_O_2_ molecule as well as the exo-cyclic O-atom of a second C_4_H_6_O_2_ molecule. In turn, each C_4_H_6_O_2_ molecule is terminally bonded to one Na^+^ via the endo-cyclic O-atom and µ-bridging to Na^+^ ions through the exo-cyclic O-atom. This results in (Na^+^)_2_ dimers (Figure 7) bridged by two C_4_H_6_O_2_ molecules and two **5** anions (through two isocarbonyl linkages each), with four further **5** anions acting as terminal isocarbonyl ligands. The so formed {Na_2_(**5**)_6_(C_4_H_6_O_2_)_2_}^4−^ units are bonded via isocarbonyl linkages to thirty further Na^+^ ions, resulting in a 3-D network (Figure S20 in the Supporting Information).

**Figure 8:**
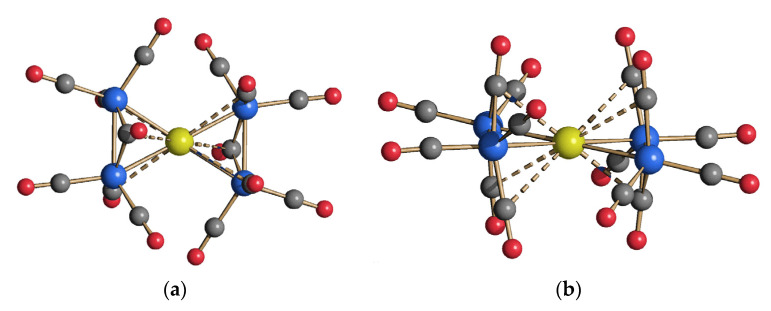
Two views of the molecular structure of the (**a**,**b**) [Au{Co_2_(CO)_7_}_2_]^−^ (**6**) anion as found in Na_2_(**7**)(**6**)·6H_2_O. Sub Van der Waals Au**···**C(O) contacts are represented as fragmented lines (yellow, Au; blue, Co; red, O; grey, C). Selected bond distances (Å): Au-Co 2.5235(7) and 2.5579(7); Co-Co 2.4848(11).

In the attempt to prepare neutral Au-Co(CO)_4_ species related to M_4_{Co(CO)_4_}_4_ (M = Cu, Ag) and {CuCo(CO)_4_}_∞_ [36,37,38], the reactions of **1** with increasing amounts of Au(I) salts was investigated. By employing a 1:1 molar ratio, the IR spectra clearly indicated that the only species present in solution was still the 2:1 adduct **4**. Even increasing the amount of Au(I) salt, the only species detected by IR spectroscopy was **4** accompanied by decomposition to Au metal. During all these attempts, among the decomposition products of the reaction, crystals of Na_2_[Au{Co_3_(CO)_9_}_2_][Au{Co_2_(CO)_7_}]·6H_2_O (Na_2_(**7**)(**6**)·6H_2_O) were obtained. This salt contains the unprecedented anions [Au{Co_2_(CO)_7_}]^−^ (**6**) and [Au{Co_3_(CO)_9_}_2_]^−^ (**7**). We may view **6** as being composed by an Au(III) center coordinated to two [Co_2_(CO)_7_]^2−^ anions (Figure 8), and its structure is reminiscent of [Au{Fe_2_(CO)_8_}]^−^ [49]. The structure of the free anion [Co_2_(CO)_7_]^2−^ has not been reported in the literature, but several of its adducts with main group and transition metals have been structurally characterized [50,51,52]. In agreement with the +3 oxidation state, the Au center is perfectly square planar.

**Figure 9:**
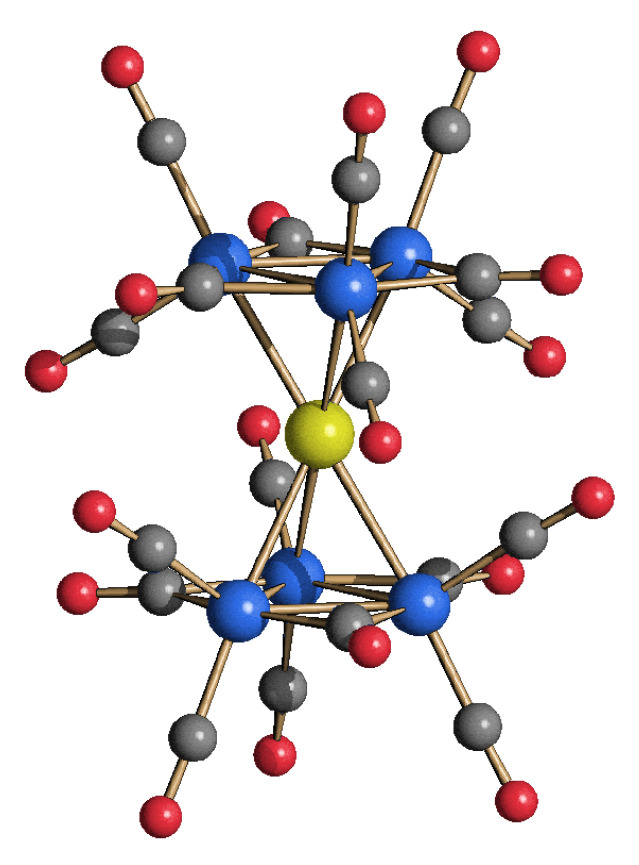
Molecular structure of the [Au{Co_3_(CO)_7_}_9_]^−^ (**7**) anion as found in Na_2_(**7**)(**6**)·6H_2_O. Sub Van der Waals Au**···**C(O) contacts are represented as fragmented lines (yellow, Au; blue, Co; red, O; grey, C). Selected bond distances (Å): Au-Co 2.6284(6), 2.6307(7) and 2.6544(8); Co-Co 2.4597(10), 2.4706(11) and 2.4799(10).

In contrast, **7** may be viewed as composed of an Au(I) center coordinated to two [Co_3_(CO)_9_]^−^ anions, acting as triply bridging ligands (Figure 9). Indeed, the coordination of Au is perfectly linear by considering the centroids of the two Co_3_ triangles. The Au-Co distances in **7** [2.6284(6), 2.6307(7) and 2.6544(8) Å] are longer than in **6** [2.5235(7) and 2.5579(7) Å] in view of the higher coordination number. The [Co_3_(CO)_9_]^−^ anion is an unsaturated cluster that possesses 46 cluster valence electrons (CVE) instead of 48 CVE as expected for a triangular cluster. This unsaturated anion has not been reported previously, whereas its conjugated hydride HCo_3_(CO)_9_ has been structurally characterized [53], as well as the related 46 CVE species HCo_3_(CO)_6_(PPh_3_)_3_ [54] and HCo_3_(CO)_3_(PMe_3_)_6_ [55,56]. In addition, the saturated 48 CVE [Co_3_(CO)_10_]^−^ anion has been also described [57].

**Figure 10:**
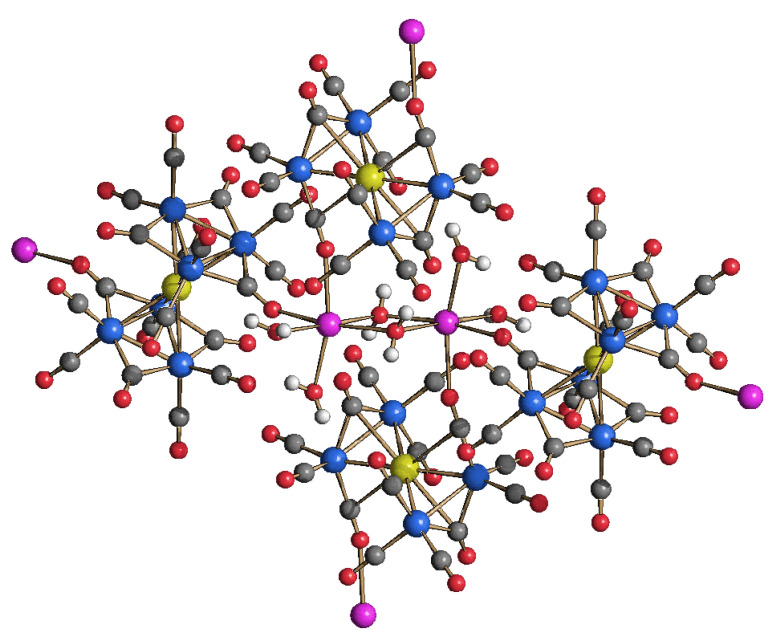
View of the Na^+^ coordination and isocarbonyl linkages present in the crystal packing of Na_2_(**7**)(**6**)·6H_2_O (yellow, Au; blue, Co; red, O; grey, C; white, H; purple, Na).

Within the crystal of Na_2_(**7**)(**6**)·6H_2_O, each Na^+^ cation is octahedrally coordinated to two O-atoms of two CO ligands one belonging to **6** and one to **7**, and four H_2_O molecules. The two isocarbonyls are in relative *cis* position, and two *cis* H_2_O molecules act as bridging ligands toward a second (equivalent) Na^+^ ion. This results in the formation of {Na_2_(**6**)_2_(**7**)_2_(H_2_O)_6_}^2−^ units (Figure 10) which are bonded via isocarbonyl linkages to four further Na^+^ ions resulting in a 2-D network. H-bonds involving H_2_O molecules and CO ligands are present in the crystals (Figure S21 and Table S4 in the Supporting Information).

Aiming at preparing Ag compounds related to **6** and **7**, the reactions of **1** with increasing amounts of Ag(I) salts were investigated. Unfortunately, these resulted only in decomposition products, among which a few crystals of [NMe_3_(CH_2_Ph)]_2_[Co_6_(CO)_15_] ([NMe_3_(CH_2_Ph)]_2_(**8**)) and [PPN]_2_[Co(THF)_4_(BF_4_)_2_][BF_4_]_2_·4CH_2_Cl_2_ ([PPN]_2_(**9**)[BF_4_]_2_·4CH_2_Cl_2_) were obtained.

[NMe_3_(CH_2_Ph)]_2_(**8**) contains the octahedral anion **8** (Figure S22 in the Supporting Information) which has been previously described [58]. Conversely, [PPN]_2_(**9**)[BF_4_]_2_·4CH_2_Cl_2_ contains the neutral Co(II) complex Co(THF)_4_(BF_4_)_2_ (Figure S23 in the Supporting Information).

## 3. Materials and Methods

### 3.1. General Experimental Procedures

All reactions and sample manipulations were carried out using standard Schlenk techniques under nitrogen and in dried solvents. All the reagents were commercial products (Aldrich, St. Louis, MO, USA) of the highest purity available and used as received, except Na[Co(CO)_4_], [PPN][Co(CO)_4_], [Cu(CH_3_CN)_4_][BF_4_] and Au(Et_2_S)Cl which were prepared according to the literature [59,60,61]. Analyses of C, H and N were obtained with a Thermo Quest Flash EA 1112NC instrument (Thermo Fisher Scientific, Waltham, MA, USA). IR spectra were recorded on a Perkin Elmer Spectrum One interferometer (Perkin Elmer, Waltham, MA, USA) in CaF_2_ cells. ESI mass spectra were recorded on a Waters Micromass ZQ4000 instrument (Waters, Milford, CT, USA) using CH_3_OH as solvent (Source Temperature = 150 °C; Capillary Voltage = 2.54 kV; Infusion Flow = 20 µL/min; Cone Voltage = 10 V). Structure drawings have been performed with SCHAKAL99 [62].

### 3.2. Synthesis of [PPN][Cu{Co(CO)_4_}_2_] ([PPN](**2**))

A solution of [Cu(CH_3_CN)_4_][BF_4_] (0.0800 g, 0.254 mmol) in THF (5 mL) was added to a solution of [PPN](**1**) (0.360 g, 0.508 mmol) in THF (10 mL) over a period of 1 h. Then, the mixture was filtered through a celite pad and the solvent removed under vacuum. The residue solid was dissolved in CH_2_Cl_2_ (10 mL) and layered with n-pentane (20 mL) affording crystals of [PPN](**2**) suitable for X-ray analyses(yield 0.186 g, 78% based on Co, 79% based on Cu).The crystals have been identified by comparison of the unit cell with that reported in the literature [41].

C_44_H_30_Co_2_CuNO_8_P_2_ (942.94): calcd. (%): C 55.99, H 3.21, N 1.49; found: C 56.12, H 3.07, N 1.71. IR (CH_2_Cl_2_, 293 K) ν_CO_: 2025(s), 1949(vs) cm^−1^.

### 3.3. Synthesis of [NEt_4_][Cu{Co(CO)_4_}_2_] ([NEt_4_](**2**))

[Cu(CH_3_CN)_4_][BF_4_] (0.429 g, 1.37 mmol) was added as a solid to a solution of Na(**1**) (0.530 g, 2.73 mmol) in CH_3_OH (10 mL). The mixture was stirred at room temperature under inert atmosphere for 1 h and then filter through a celite pad. Then, a saturated solution of [NEt_4_]Br in H_2_O (30 mL) was added up to complete precipitation. The resulting solid was washed with H_2_O (40 mL) and extracted in CH_3_CN (15 mL). The solvent was removed under reduced pressure and the residue dissolved in CH_2_Cl_2_ (10 mL). Crystals of [NEt_4_](**2**) suitable for X-ray analyses were obtained by layering n-pentane (20 mL) on the CH_2_Cl_2_ solution (yield 0.601 g, 82% based on Co, 82% based on Cu).

C_16_H_20_Co_2_CuNO_8_ (535.73): calcd. (%): C 35.89, H 3.77, N 2.62; found: C 35.64, H 3.98, N 2.88. IR (CH_2_Cl_2_, 293 K) ν_CO_: 2026(s), 1945(vs) cm^−1^. IR (nujol, 293 K) ν_CO_: 2025(s), 1952(m), 1922(ms), 1865(w) cm^−1^.

### 3.4. Synthesis of [NEt_4_][Ag{Co(CO)_4_}_2_] ([NEt_4_](**3**))

AgNO_3_ (0.283 g,1.68 mmol) was added as a solid to a solution of Na(**1**) (0.650 g, 3.35 mmol) in CH_3_OH (10 mL). The mixture was stirred at room temperature under inert atmosphere and the reaction was monitored by FT-IR spectroscopy. After 1 h the mixture was filtered through a celite pad and then the product was precipitated by adding a saturated solution of [NEt_4_]Br in H_2_O (20 mL). The solid was collected by filtration, washed with H_2_O (40 mL) and extracted in CH_3_CN (15 mL). The yellow solution was evaporated to dryness at reduced pressure, dissolved in CH_2_Cl_2_(10 mL) and layered with n-pentane (20 mL) affording crystals of [NEt_4_](**3**) suitable for X-ray analyses (yield 0.662 g, 68% based on Co, 68% based on Ag).

C_16_H_20_AgCo_2_NO_8_ (580.06): calcd. (%): C 33.17, H 3.48, N 2.42; found: C 32.87, H 3.19, N 2.75. IR (CH_2_Cl_2_, 293 K) ν_CO_: 2026(s), 1938(vs) cm^−1^. IR (nujol, 293 K) ν_CO_: 2023(s), 1947(ms), 1904(ms) cm^−1^.

### 3.5. Synthesis of [PPN][Au{Co(CO)_4_}_2_] ([PPN](**4**))

A solution of Au(Et_2_S)Cl (0.099 g, 0.307 mmol) in THF (5 mL) was added to a solution of [PPN](**1**) (0.290 g, 0.409 mmol) in THF (10 mL) over a period of 1 h at room temperature under nitrogen atmosphere. Then, the mixture was filtered through a celite pad and the solvent removed under vacuum. The residue was dissolved in CH_2_Cl_2_ (10 mL) and layered with n-pentane (20 mL) affording crystals of [PPN](**4**) suitable for X-ray analyses (yield 0.134 g, 61% based on Co, 41% based on Au). The crystals have been identified by comparison of the unit cell with that reported in the literature [42].

C_44_H_30_AuCo_2_NO_8_P_2_ (1076.98): calcd. (%): C 49.03, H 2.81, N 1.30; found: C 48.85, H 3.09, N 1.62. IR (CH_2_Cl_2_, 293 K) ν_CO_: 2025(s), 1957(vs) cm^−1^.

### 3.6. Synthesis of [NEt_4_][Au{Co(CO)_4_}_2_] ([NEt_4_](**4**))

Au(Et_2_S)Cl (0.535 g, 1.66 mmol) was added as a solid to a solution of Na(**1**) (0.650 g, 3.35 mmol) in CH_3_OH (10 mL). The mixture was stirred at room temperature under inert atmosphere and the reaction was monitored by FT-IR spectroscopy. After 1 h the mixture was filtered through a celite pad and then the product was precipitated by adding a saturated solution of [NEt_4_]Br in H_2_O (20 mL). The solid was collected by filtration, washed with H_2_O (40 mL) and extracted in CH_3_CN (15 mL). The yellow solution was evaporated to dryness at reduced pressure, dissolved in CH_2_Cl_2_ (10 mL) and layered with n-pentane (20 mL) affording crystals of [NEt_4_](**4**) suitable for X-ray analyses (yield 0.551 g, 49% based on Co, 50% based on Au).

C_16_H_20_AuCo_2_NO_8_ (669.16):calcd. (%): C 28.70, H 3.01, N 2.09; found: C 28.91, H 2.84, N 1.78. IR (CH_2_Cl_2_, 293 K) ν_CO_: 2025(s), 1959(vs) cm^−1^.

### 3.7. Synthesis of Na_2_[Ag_2_{Co(CO)_4_}_4_]·C_4_H_6_O_2_ (Na_2_(**5**)·C_4_H_6_O_2_)

Solid AgCl (0.932 g, 6.45 mmol) was added in small portions to a THF (10 mL) solution of Na(**1**) (0.250 g, 1.29 mmol). The mixture was stirred at room temperature under inert atmosphere and the reaction was monitored by FT-IR spectroscopy. After 36h the mixture was filtered through a celite pad and then, the pale-yellow solution was evaporated to dryness at reduced pressure. The residue was dissolved in CH_2_Cl_2_ (10 mL). Suitable crystal for X-ray diffraction of Na_2_(**5**)·C_4_H_6_O_2_ were obtained by slow diffusion of n-pentane (20 mL) on the dichloromethane solution in the presence of some C_4_H_6_O_2_ (yield 0.209 g, 58% based on Co, 6% based on Ag).

C_27_H_12_Ag_2_Co_4_Na_2_O_20_ (1117.78): calcd. (%): C 28.14, H 1.05; found: C 28.32, H 0.87. IR (CH_2_Cl_2_, 293 K) ν_CO_: 2030(s), 1929(vs) cm^−1^. IR (THF, 293 K) ν_CO_: 2024(s), 1941(vs) cm^−1^.

### 3.8. Synthesis of [PPN]_2_[Ag_2_{Co(CO)_4_}_4_]·C_5_H_12_ ([PPN]_2_(**5**)·C_5_H_12_)

AgNO_3_(0.152 g, 0.897 mmol) was added as a solid, in small portions, to a solution of [PPN](**1**) (0780 g, 1.100 mmol) in CH_2_Cl_2_ (10 mL). The mixture was stirred at room temperature under nitrogen for 2h and, then, the mixture was filtered through celite and the celite pad washed with CH_2_Cl_2_ (10 mL). The clear orange solution obtained was concentrated under reduced pressure and layered with n-pentane (20 mL) affording crystals of [PPN]_2_(**5**)·C_5_H_12_ suitable for X-ray analysis (yield 0.310 g, 55% based on Co, 34% based on Ag).

C_93_H_72_Ag_2_Co_4_N_2_O_16_P_4_ (2048.86): calcd. (%): C 54.55, H 3.55, N 1.37; found: C 54.21, H 3.84, N 1.04. IR (CH_2_Cl_2_, 293 K) ν_CO_: 2026(s), 1938(vs) cm^−1^. IR (nujol, 293 K) ν_CO_: 2023(s), 1954(m), 1940(vs), 1916(m), 1883(w) cm^−1^.

### 3.9. Synthesis of [NBu_4_]_2_[Ag_2_{Co(CO)_4_}_4_] ([NBu_4_]_2_(**5**))

AgNO_3_ (0.283 g, 1.68 mmol) was added as a solid to a solution of Na(**1**) (0.650 g, 3.35 mmol) in CH_3_OH (10 mL). The mixture was stirred at room temperature under inert atmosphere and the reaction was monitored by FT-IR spectroscopy. After 1 h the mixture was filtered through a celite pad and then the product was precipitated by adding a saturated solution of [NBu_4_]Br in H_2_O (20 mL). The solid was collected by filtration, washed with H_2_O (40 mL) and extracted in CH_3_CN (15 mL). The yellow solution was evaporated to dryness at reduced pressure, dissolved in CH_2_Cl_2_ (10 mL) and layered with n-pentane (20 mL) affording crystals of [NBu_4_](**5**) suitable for X-ray analyses (yield 0.721 g, 62% based on Co, 62% based on Ag).

C_48_H_72_Ag_2_Co_4_N_2_O_16_ (1384.53): calcd. (%): C 41.68, H 5.25, N 2.03; found: C 41.40, H 5.39, N 1.84. IR (CH_2_Cl_2_, 293 K) ν_CO_: 2028(s), 1941(vs) cm^−1^.

### 3.10. Synthesis of [NMe_4_]_2_[Ag_2_{Co(CO)_4_}_4_] ([NMe_4_]_2_(**5**))

AgNO_3_ (0.283 g, 1.68 mmol) was added as a solid to a solution of Na(**1**) (0.650 g, 3.35 mmol) in CH_3_OH (10 mL). The mixture was stirred at room temperature under inert atmosphere and the reaction was monitored by FT-IR spectroscopy. After 1 h the mixture was filtered through a celite pad and then the product was precipitated by adding a saturated solution of [NMe_4_]Cl in H_2_O (20 mL). The solid was collected by filtration, washed with H_2_O (40 mL) and extracted in CH_3_CN (15 mL). The yellow solution was evaporated to dryness at reduced pressure, dissolved in CH_2_Cl_2_ (10 mL) and layered with n-pentane (20 mL) affording crystals of [NMe_4_](**5**) suitable for X-ray analyses (yield 0.526 g, 60% based on Co, 60% based on Ag).

C_24_H_24_Ag_2_Co_4_N_2_O_16_ (1047.91): calcd. (%): C 27.54, H 2.31, N 2.68; found: C 27.29, H 2.64, N 2.88. IR (CH_2_Cl_2_, 293 K) ν_CO_: 2027(s), 1938(vs) cm^−1^. IR (nujol, 293 K) ν_CO_: 2025(s), 1974(m), 1935(vs), 1913(m), 1885(w) cm^−1^.

### 3.11. Synthesis of Na_2_[Au{Co_3_(CO)_9_}_2_][Au{Co_2_(CO)_7_}_2_]·6H_2_O (Na_2_(**7**)(**6**)·6H_2_O)

A solution of Au(Et_2_S)Cl (0.112 g, 0.348 mmol) in CH_2_Cl_2_ (5 mL) was added to a solution of Na(**1**) (0.270 g, 1.39 mmol) in CH_2_Cl_2_ (10 mL) over a period of 1 h. The mixture was stirred at room temperature under inert atmosphere. At the end of the reaction, the mixture was filtered, and the dark green dichloromethane solution was layered with n-pentane (30 mL). Black crystals of Na_2_(**7**)(**6**)·6H_2_O were obtained from the CH_2_Cl_2_/pentane double layer as a decomposition product of the reaction(yield 0.062 g, 22% based on Co, 9% based on Au).

C_32_H_12_Au_2_Co_10_Na_2_O_38_ (2033.63): calcd. (%): C 18.89, H 0.59; found: C 20.10, H 1.12. IR (CH_2_Cl_2_, 293 K) ν_CO_: 2031(s), 1967(vs) cm^−1^. IR (nujol, 293 K) ν_CO_: 2072(m), 2021(s), 1988(w), 1968(w) cm^−1^.

### 3.12. Synthesis of [NMe_3_(CH_2_Ph)]_2_[Co_6_(CO)_15_] ([NMe_3_(CH_2_Ph)]_2_(**8**))

AgNO_3_ (1.07 g, 6.36 mmol) was added as a solid to a solution of Na(**1**) (0.650 g, 3.35 mmol) in CH_3_OH (10 mL). The mixture was stirred at room temperature under inert atmosphere and the reaction was monitored by FT-IR spectroscopy. After 1 h the mixture was filtered through a celite pad and then the product was precipitated by adding a saturated solution of [NMe_3_(CH_2_Ph)]Cl in H_2_O (20 mL). The solid was collected by filtration, washed with H_2_O (40 mL) and extracted in CH_3_CN (15 mL). The yellow solution was evaporated to dryness at reduced pressure, dissolved in CH_2_Cl_2_ (10 mL) and layered with n-pentane (20 mL). A few crystals of [NMe_3_(CH_2_Ph)]_2_(**8**) were isolated from the CH_2_Cl_2_/pentane double layer as a decomposition product of the reaction. These were analyzed by SC-XRD but, owing the very limited amount, no further analysis was carried out.

### 3.13. Synthesis of [PPN]_2_[Co(THF)_4_(BF_4_)_2_][BF_4_]_2_·4CH_2_Cl_2_ ([PPN]_2_(**9**)[BF_4_]_2_·4CH_2_Cl_2_)

AgBF_4_ (0.231 g,1.18 mmol) was added as a solid, in small portions, to a solution of [PPN](**1**) (0.280 g, 0.395 mmol) in THF (10 mL).The mixture was stirred at room temperature under nitrogen for 2h. Then, the mixture was filtered through celite and the celite pad washed with THF (5 mL). The solution was evaporated to dryness under reduced pressure and the residue dissolved in CH_2_Cl_2_ (10 mL). A few crystals of [PPN]_2_[Co(THF)_4_(BF_4_)_2_][BF_4_]_2_·4CH_2_Cl_2_were isolated from the CH_2_Cl_2_/pentane double layer as a decomposition product of the reaction. These were analyzed by SC-XRD but, owing the very limited amount, no further analysis was carried out.

### 3.14. X-ray Crystallographic Study

Crystal data and collection details for [NEt_4_](**2**), [NEt_4_](**3**), [NEt_4_](**4**), Na_2_(**5**)·C_4_H_6_O_2_, [PPN]_2_(**5**)·C_5_H_12_, [NBu_4_]_2_(**5**), [NMe_4_]_2_(**5**), Na_2_(**7**)(**6**)·6H_2_O, [NMe_3_(CH_2_Ph)]_2_(**8**) and [PPN]_2_(**9**)[BF_4_]_2_·4CH_2_Cl_2_ are reported in Table S5 in the Supporting Information. The diffraction experiments were carried out on a Bruker APEX II diffractometer equipped with a PHOTON2 detector using Mo–Kα radiation. Data were corrected for Lorentz polarization and absorption effects (empirical absorption correction SADABS) [63]. Structures were solved by direct methods and refined by full-matrix least-squares based on all data using *F*^2^ [64]. Hydrogen atoms were fixed at calculated positions and refined by a riding model. All non-hydrogen atoms were refined with anisotropic displacement parameters.

### 3.15. Computational Details

All density functional theory (DFT) calculations were carried out using the Gaussian 16 package [65] and the B3LYP functional [66,67,68].

Geometry optimizations were performed using LANL2DZ basis set with pseudpotential for transition metals [69], whereas 6-31G(d,p) basis set was used for all other atoms [70], confirming the character of the stationary points by vibrational analysis. IR frequencies have been computed analytically, as implemented in Gaussian 16, and rescaled using a 0.961 scaling factor [71].

For the thermodynamics of complexes’ equilibria, the reported Gibbs free energies have been calculated using larger-basis-set (i.e., 6-311+G(2d,2p) for all atoms but transition metals) single-point computations and including Gibbs free energy corrections (at 298.15 K) and Grimme-D3 corrections for dispersions [72] and using the conductor like polarizable continuum model (C-PCM) [73,74] for solvation effects.

## 4. Conclusions

The M-Co(CO)_4_ (M = Cu, Ag, Au) system has been reinvestigated unraveling a new example of polymerization isomerism in metal carbonyl clusters. Thus, depending on the crystallization conditions, the monomer **3** or the dimer **5** have been isolated in the solid state in the case of Ag. Conversely, only the monomers **2** and **4** have been obtained for Cu and Au, respectively. This difference relies on thermodynamic effects, as pointed out by DFT calculations. Several other examples of isomerism in metal carbonyl clusters, molecular clusters and nanoclusters have been described in the literature as summarized in the introduction. The scope of this field is rapidly expanding and gives new insights into isomerism, which for a longtime has mainly been discussed within the framework of organic and coordination chemistry.

## Figures and Tables

**Table 1:** Main bond distances (Å) and angles (°) for [Cu{Co(CO)_4_}_2_]^−^ (**2**), [Ag{Co(CO)_4_}_2_]^−^ (**3**), [Au{Co(CO)_4_}_2_]^−^ (**4**) and [Ag_2_{Co(CO)_4_}_4_]^2−^ (**5**).

	2 ^a^	3 ^b^	4 ^c^	5 ^d^	5 ^e^	5 ^f^	5 ^g^
**M-Co_t_^h^**	2.393(3)–2.411(3) Av. 2.402(6)	2.4392(17)–2.4450(17) Av. 2.442(5)	2.363(5)–2.517(6) Av. 2.449(12)	2.6537(3)	2.6692(7)	2.67936(18)	2.669(4) and 2.679(4)
**M-Co_b_^h^**	-	-	-	2.8576(3)	2.8094(10)	2.75393(19) and 2.8475(2)	2.787(4)–2.805(4) Av. 2.798(8)
**M-M**	-	-	-	2.8425(3)	2.8775(7)	2.91970(17)	2.916(4) and 2.923(4)
**Co_t_-M-Co_t_^h^**	180.0	180	180	-	-	-	
**Co_t_-M-M**	-	-	-	174.595(9)	177.210(13)	171.915(6)	175.50(16) and 177.28(15)
**M-Co_b_-M ^h^**	-	-	-	60.725(7)	61.951(12)	62.806(4)	63.08(11) and 62.75(11)
**Co_b_-M-Co_b_^h^**	-	-	-	119.275(7)	118.048(12)	117.194(5)	116.93(11) and 117.25(11)
**Co_b_-M-M ^h^**	-	-	-	61.273(7)	59.51(2)	57.031(5) and 60.163(5)	58.20(10) and 58.47(11)

^a^ As found in [NEt_4_](**2**). ^b^ As found in [NEt_4_](**3**). ^c^ As found in [NEt_4_](**4**). ^d^ As found in Na_2_(**5**)·C_4_H_6_O_2_. ^e^ As found in [PPN]_2_(**5**)·C_5_H_12_. ^f^ As found in [NBu_4_]_2_(**5**). ^g^ As found in [NMe_4_]_2_(**5**). ^h^ Co_t_ refers to Co(CO)_4_ groups bonded to a single M (terminal position), whereas Co_b_ refers to Co(CO)_4_ groups bonded to two M atoms (bridging position).

## Data Availability

Data are available from the authors.

## Supplementary Materials

The following are available online. DFT optimized structures; experimental and simulated IR spectra; ESI-MS spectra; crystal packings of Na_2_(**5**)·C_4_H_6_O_2_ and Na_2_(**7**)(**6)**·6H_2_O, H-bonds of Na_2_(**7**)(**6**)·6H_2_O; molecular structures of **8** and **9**; X-ray Crystallographic Study. CCDC reference numbers 2063733-2063742 contain the supplementary crystallographic data for the X-ray studies reported in this paper. These data can be obtained free of charge at www.ccdc.cam.ac.uk/conts/retrieving.html (or from the Cambridge Crystallographic Data Centre, 12, Union Road, Cambridge CB2 1EZ, UK; fax: (internat.) +44-1223/336-033; e-mail: deposit@ccdc.cam.ac.uk).

## Sample Availability

Samples of the compounds **2**–**5** are available from the authors.
