# Supplementary material for: Polymerization Isomerism in Co-M (M = Cu, Ag, Au) Carbonyl Clusters: Synthesis, Structures and Computational Investigation
Source: Molecules. 2021 Mar 11;26(6):1529. doi: 10.3390/molecules26061529 (PMC7998641; doi:10.3390/molecules26061529)
Supplement: Supplementary file 1 [file molecules-26-01529-s001.pdf]

Polymerization Isomerism in Co-M (M = Cu, Ag, Au) Carbonyl Clusters: Synthesis, Structures and Computational Investigation

Cristiana Cesari, Beatrice Berti, Francesco Calcagno, Cristina Femoni, Marco Garavelli, Maria Carmela Iapalucci, Ivan Rivalta and Stefano Zacchini

Supporting Information

Table of contents

	Pages
**Scheme S1:** DFT computations of **3** and **5** free energies	S2
**Figure S0:** DFT computations of **2** isomers	S2
**Figures S1–S12:** Experimental and simulated IR spectra	S3
**Figures S13–S19:** ESI-MS spectra of **2-5**	S10
**Figures S20–S21:** Crystal packing of Na_2(**5**)·$C_4H_6O_2$ and Na_2[**7**][**6**]·$6H_2O$	S17
**Table S4**: H-bonds of Na_2(**7**)(**6**)·$6H_2O$	S20
**Figures S22–S23:** Molecular structures of **8** and **9**	S21
**Table S5:** X-ray Crystallographic Study	S25
DFT optimized geometries (cartesian coordinates)	S28

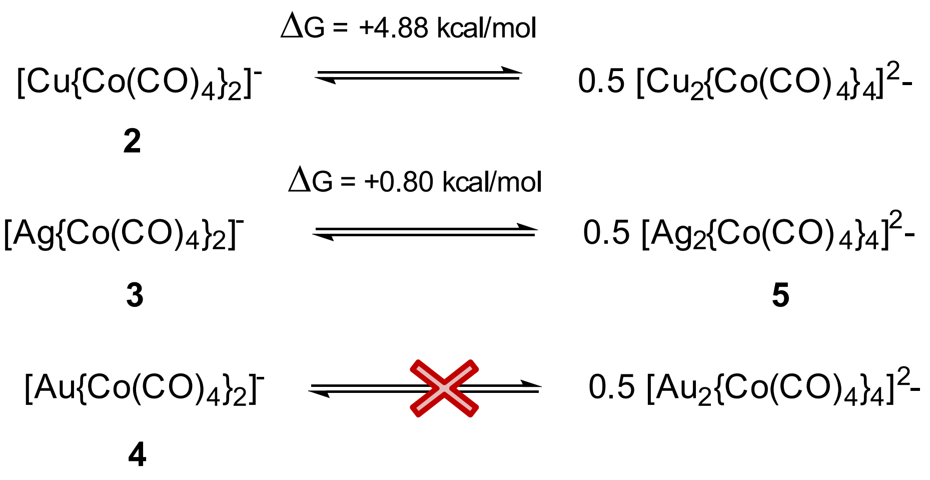

Scheme S1. Free energies differences between $[M\{Co(CO)_4\}_2]^-$ and $[M_2\{Co(CO)_4\}_4]^{2-}$, computed at B3LYP/LANL2DZ/6-311+G(2d,2p) level of theory for Ag, Cu and Au, showing a minimal energetic cost (ca. 1 kcal/mol) for dimerization in the case of Ag, a significant endergonicity for Cu (>5 kcal/mol) and a highly unstable dimer for Au (i.e. it was not possible to reach an optimized geometry without dissociation into monomers).

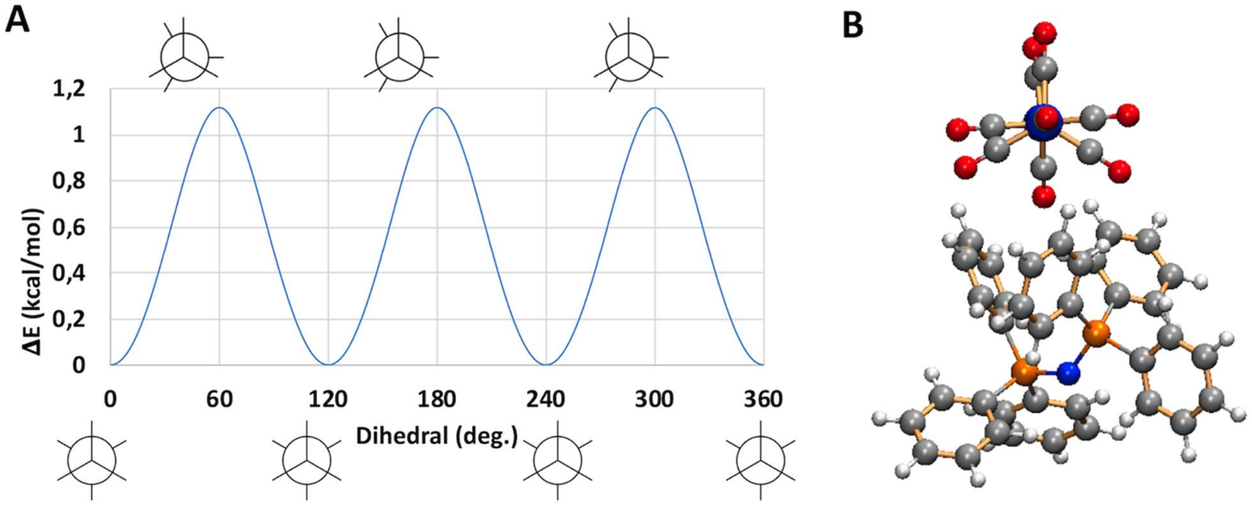

Figure S0. DFT computations of the isomers of **2** in gas-phase. (A) Potential energy (relaxed) scan of the C-Co-Cu-C dihedral for the TBP-TBP isomer showing the more stable staggered conformer with respect to the eclipsed one. (B) Optimized geometry of [PPN][**2**] salt, showing stabilization of TBP-Td isomer in presence of the counterion.

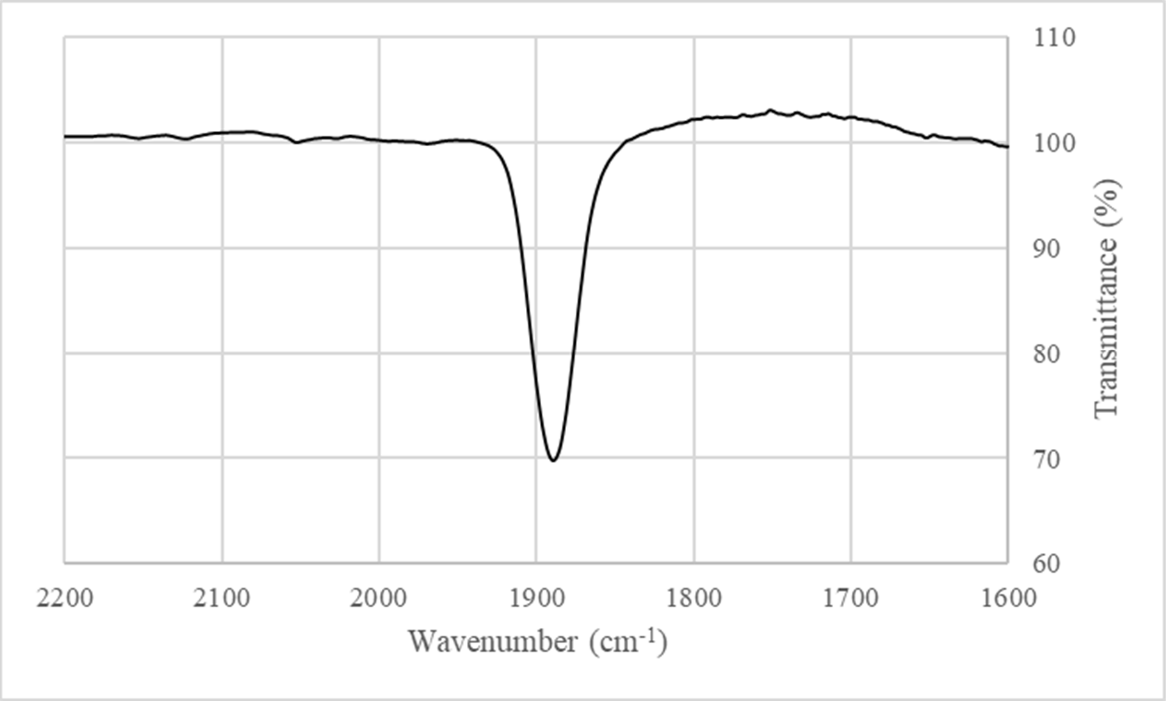

Figure S1. FT-IR spectrum of [PPN][$Co(CO)_4$] ([PPN][**1**]) in CH_2Cl_2. Spectral resolution 2 cm^{-1}.

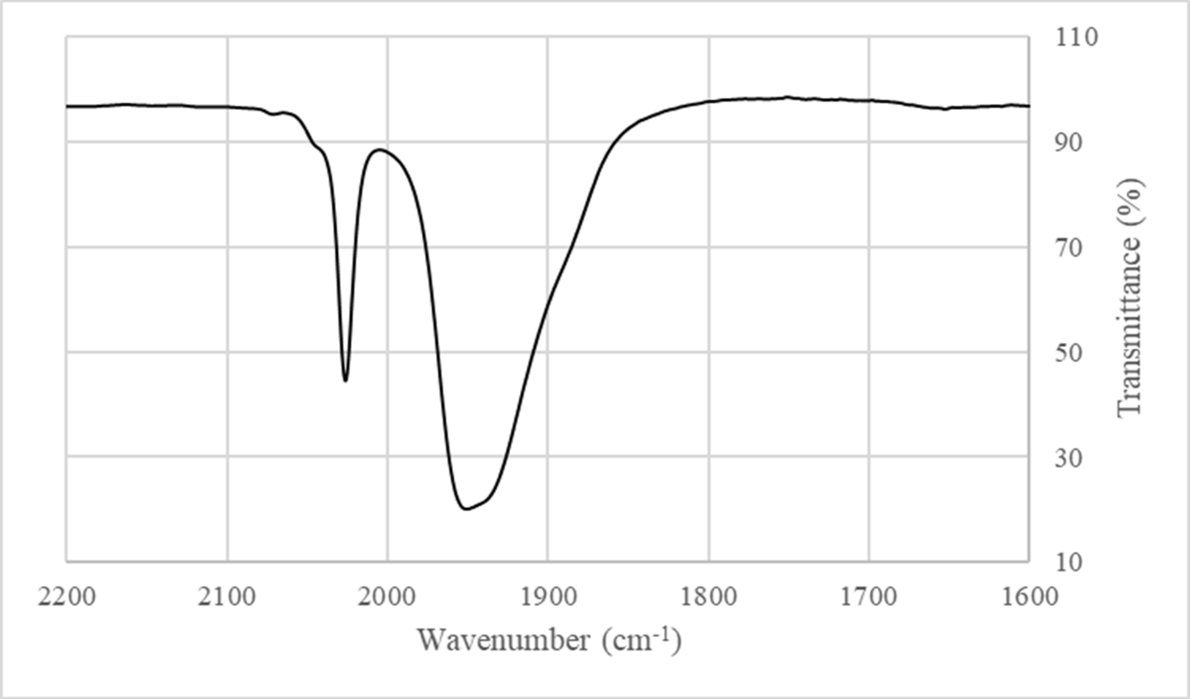

Figure S2. FT-IR spectrum of [NEt_4][Cu{$Co(CO)_4$}$_2$] ([NEt_4][**2**]) in CH_2Cl_2. Spectral resolution 2 cm^{-1}.

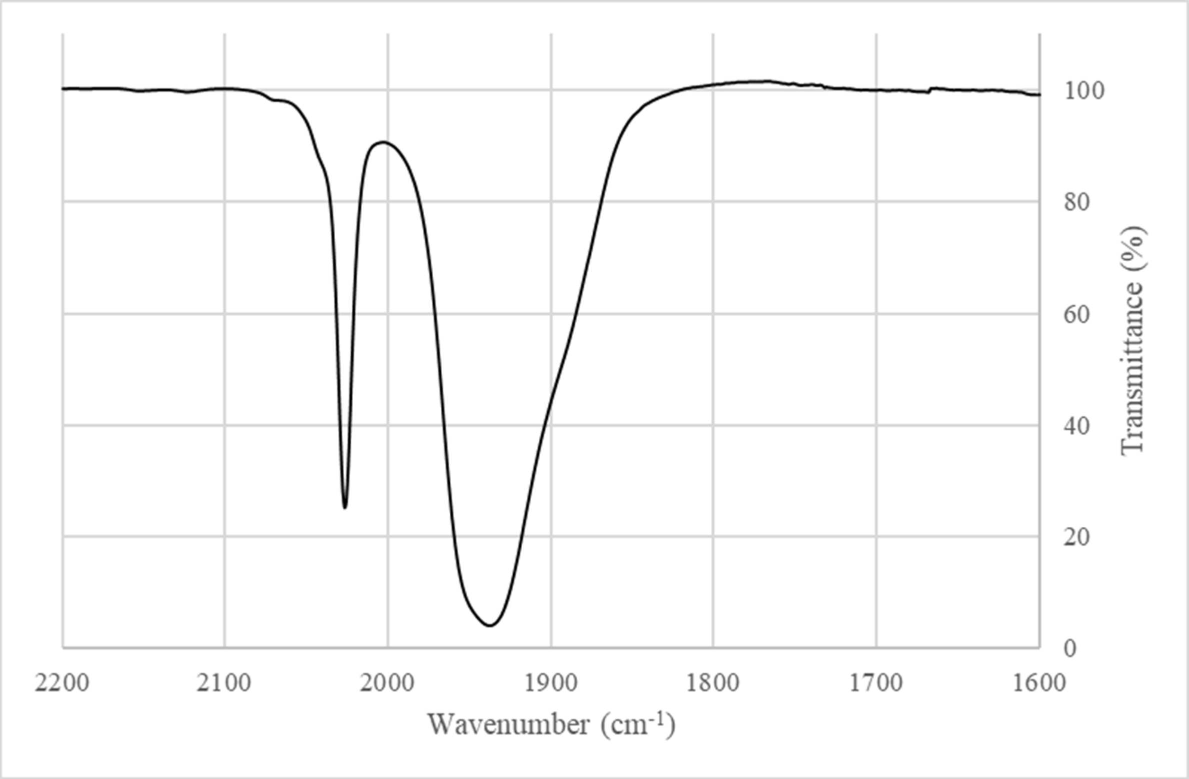

Figure S3. FT-IR spectrum of $[NEt_4][Ag\{Co(CO)_4\}_2]$ ($[NEt_4]$[**3**]) in CH_2Cl_2. Spectral resolution 2 cm^{-1}.

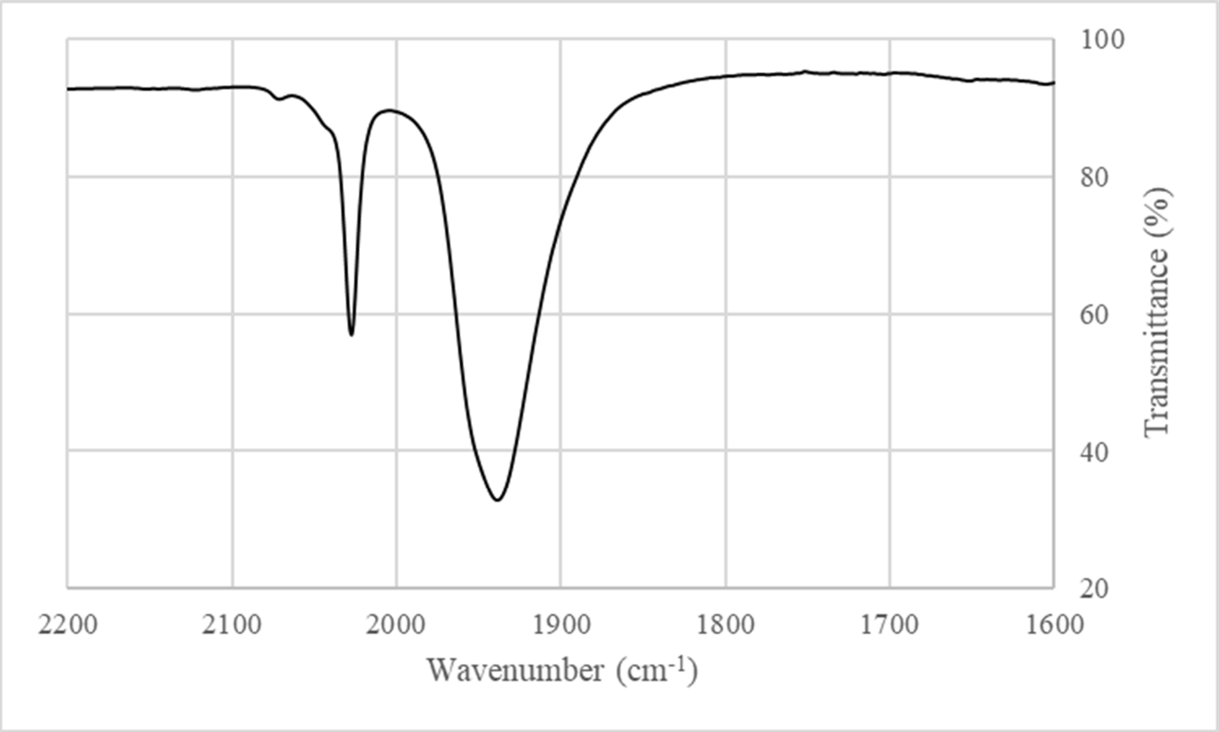

Figure S4. FT-IR spectrum of $[NMe_4]_2[Ag_2\{Co(CO)_4\}_4]$ ($[NMe_4]_2$[**5**]) in CH_2Cl_2. Spectral resolution 2 cm^{-1}.

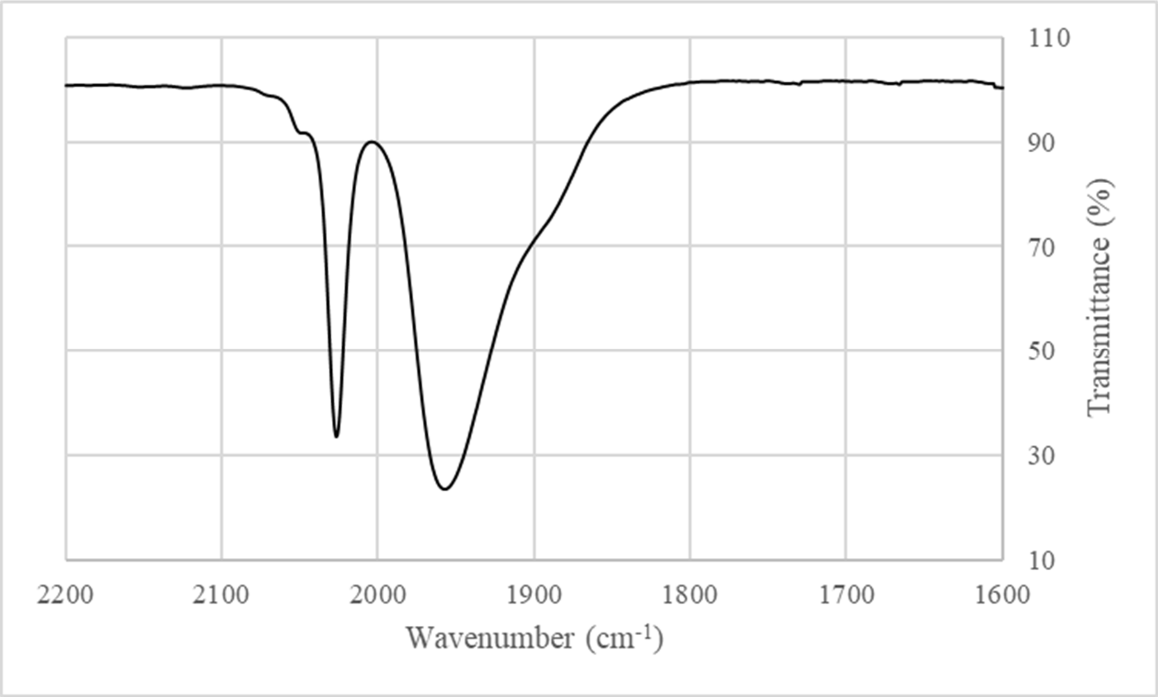

Figure S5. FT-IR spectrum of $[NEt_4][Au\{Co(CO)_4\}_2]$ ($[NEt_4]$[**4**]) in CH_2Cl_2. Spectral resolution 2 cm^{-1}.

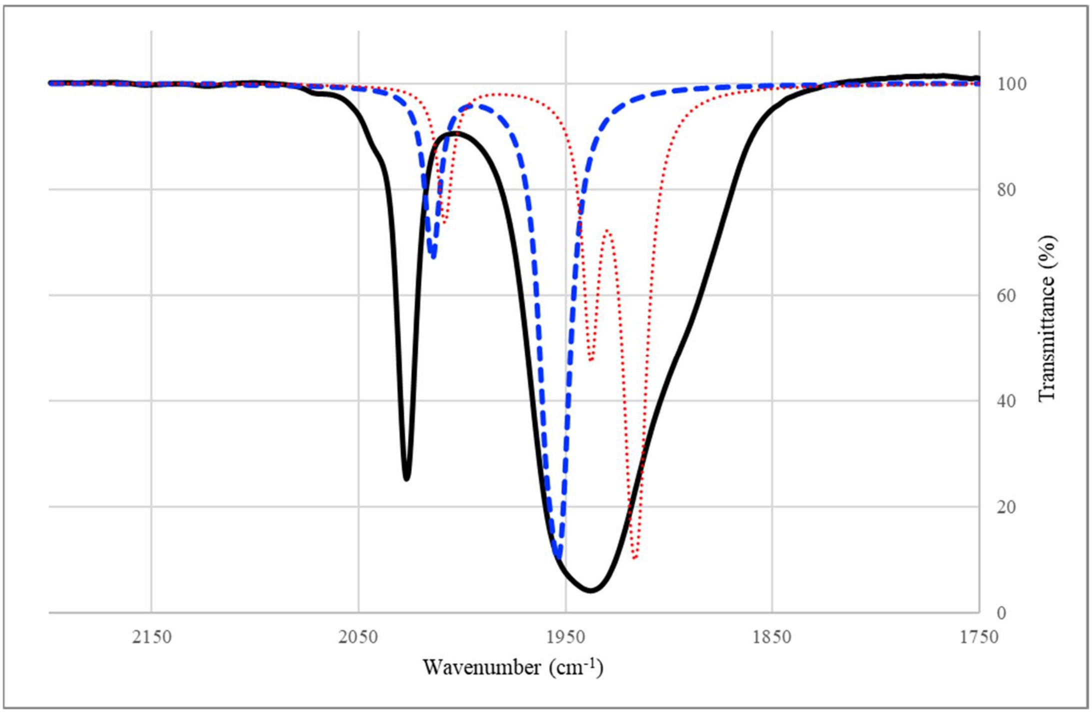

Figure S6. IR spectra of $[NEt_4][Ag\{Co(CO)_4\}_2]$ ($[NEt_4]$[**3**]) in CH_2Cl_2. Experimental spectral (black line) resolution 2 cm^{-1}. DFT-B3LYP/LANL2DZ/6-31G(d,p) simulated spectra of **3** in gas-phase (blue dashed line) and in presence of the CH_2Cl_2 implicit solvent (red dashed line).

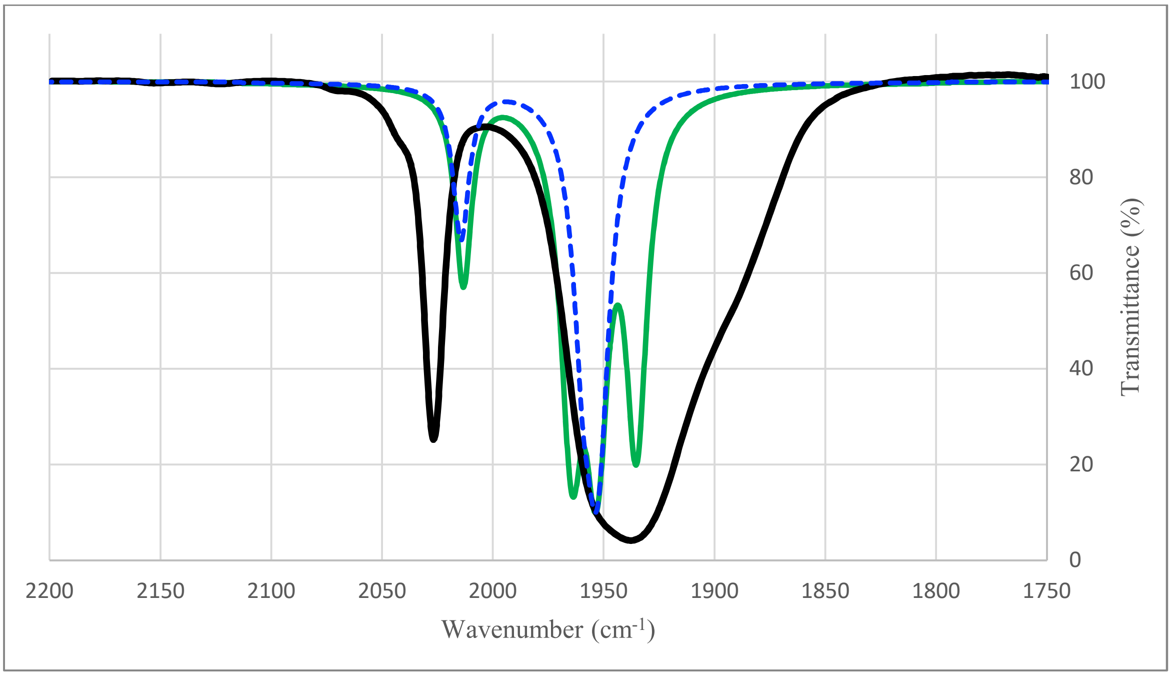

Figure S7. IR spectra of $[NEt_4][Ag\{Co(CO)_4\}_2]$ ($[NEt_4]$[**3**]) in CH_2Cl_2. Experimental spectral (black line) resolution 2 cm^{-1}. DFT-B3LYP/LANL2DZ/6-31G(d,p) simulated spectra of **3** in

gas-phase (blue dashed line) and in presence of two explicit molecules of CH_2Cl_2 solvent (green line).

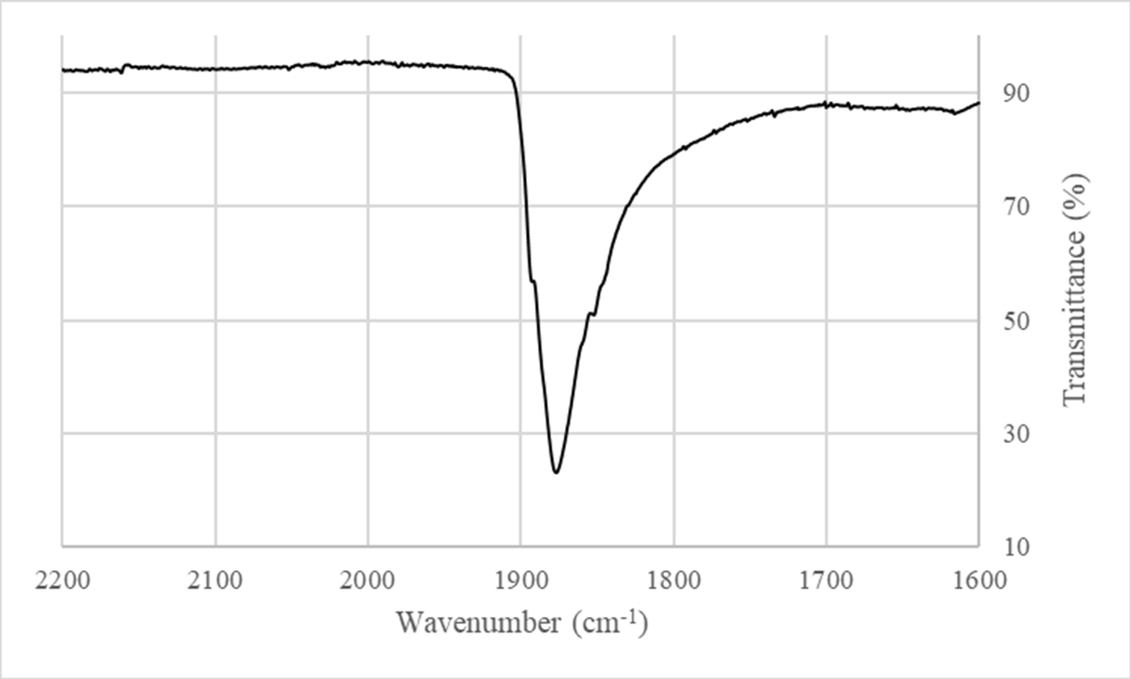

Figure S8. FT-IR-ATR spectrum of [PPN][$Co(CO)_4$] ([PPN][**1**]) as solid. Spectral resolution 2 cm^{-1}.

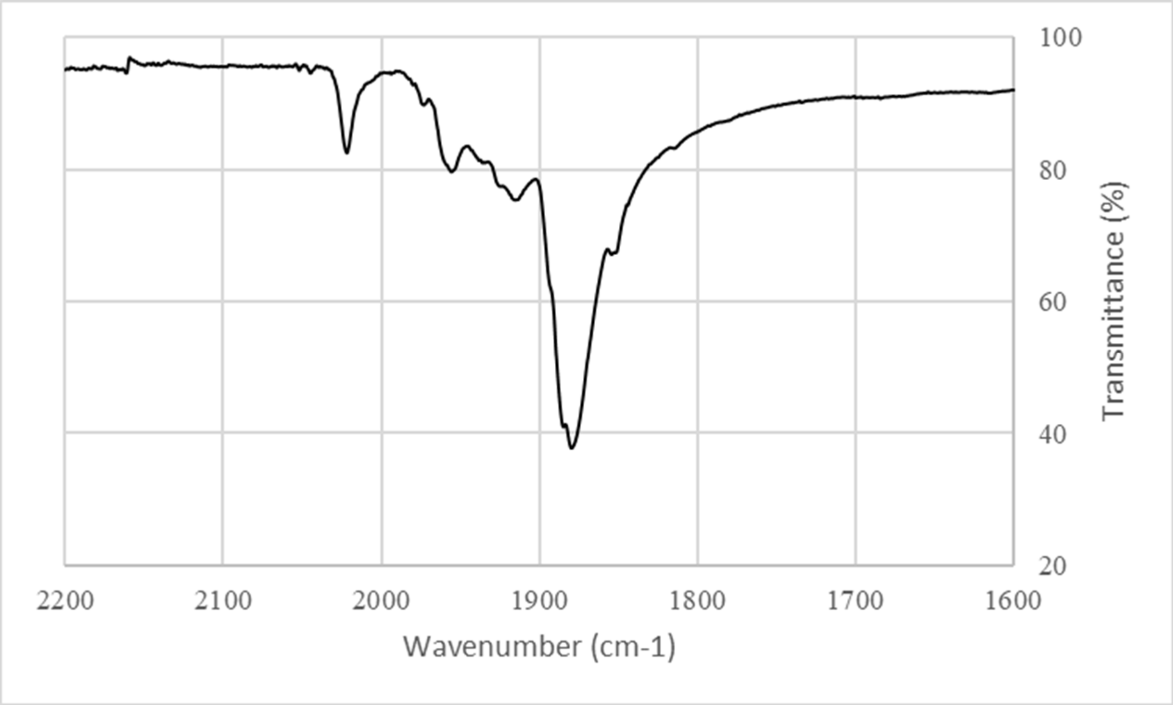

Figure S9. FT-IR-ATR spectrum of $[NEt_4][Cu\{Co(CO)_4\}_2]$ ($[NEt_4]$[**2**]) as solid. Spectral resolution 2 cm^{-1}.

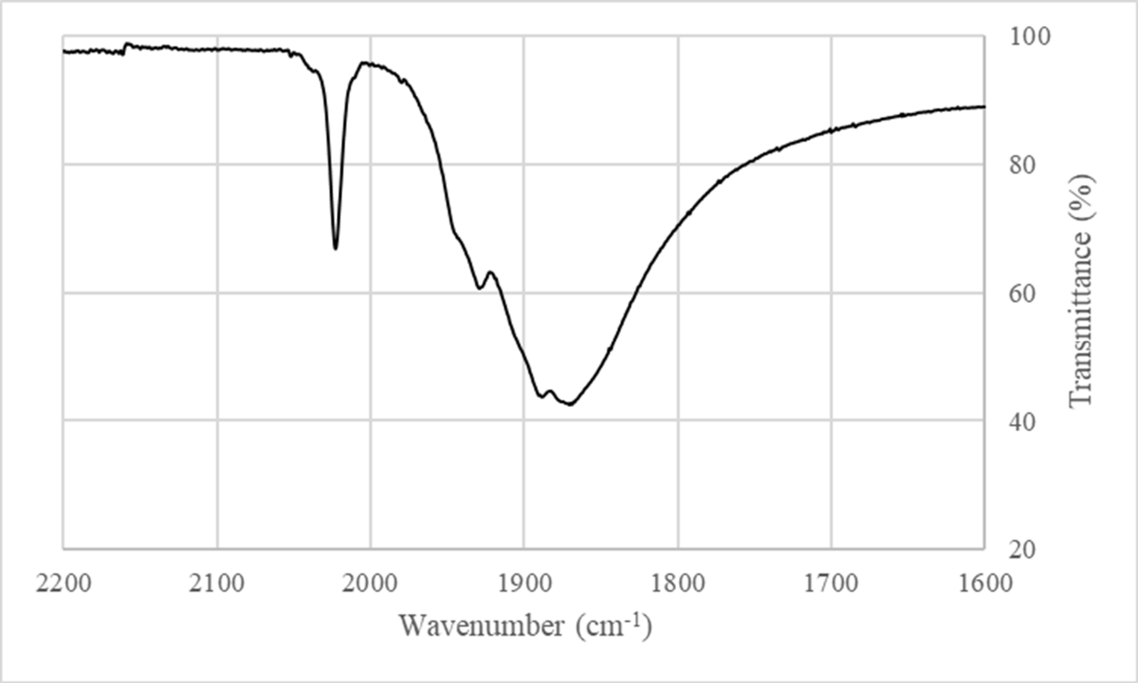

Figure S10. FT-IR-ATR spectrum of $[NEt_4][Ag\{Co(CO)_4\}_2]$ ($[NEt_4]$[**3**]) as solid. Spectral resolution 2 cm^{-1}.

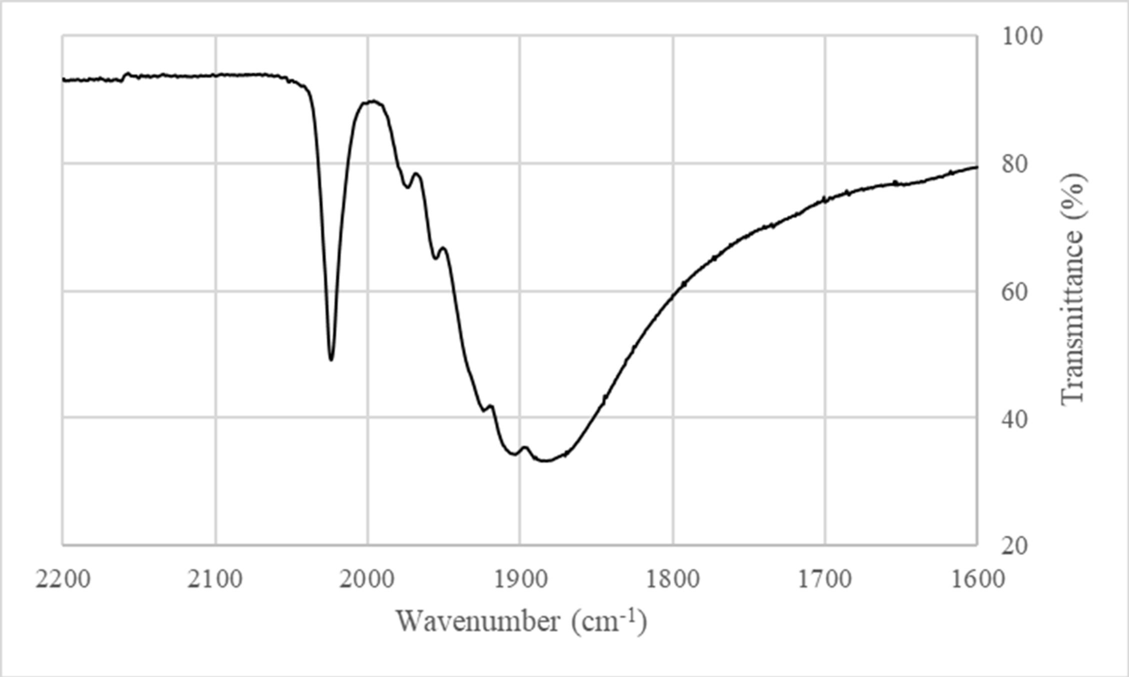

Figure S11. FT-IR-ATR spectrum of $[NMe_4]_2[Ag_2\{Co(CO)_4\}_4]$ ($[NMe_4]_2$[**5**]) as solid. Spectral resolution 2 cm^{-1}.

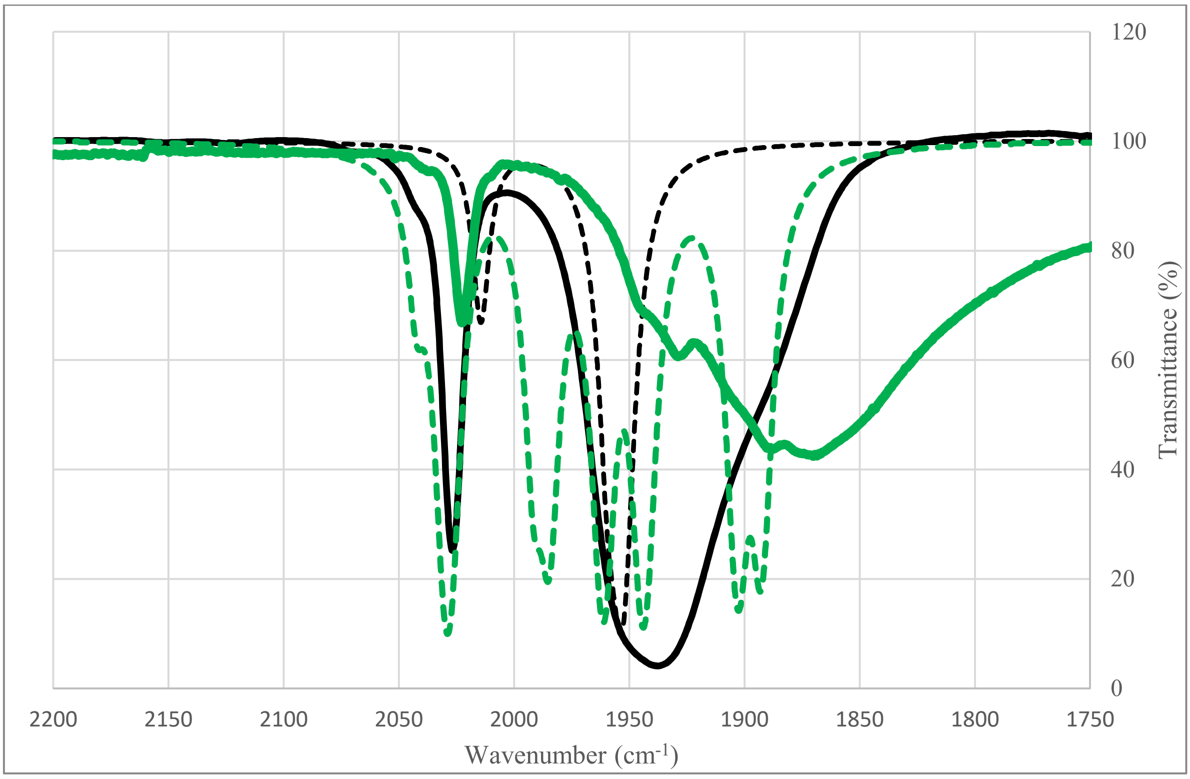

Figure S12. FT-ATR spectra of $[NEt_4][Ag\{Co(CO)_4\}_2]$ ($[NEt_4]$[**3**]) (green solid line) and IR spectra of in CH_2Cl_2 (black solid line). Experimental spectral resolution 2 cm^{-1}. DFT-B3LYP/LANL2DZ/6-31G(d,p) simulated spectra of **3** in gas-phase (black dashed line) and in presence of an explicit molecule of the $[NEt_4]^+$ counter-ion in a tight ion-pair conformation (green dashed line).

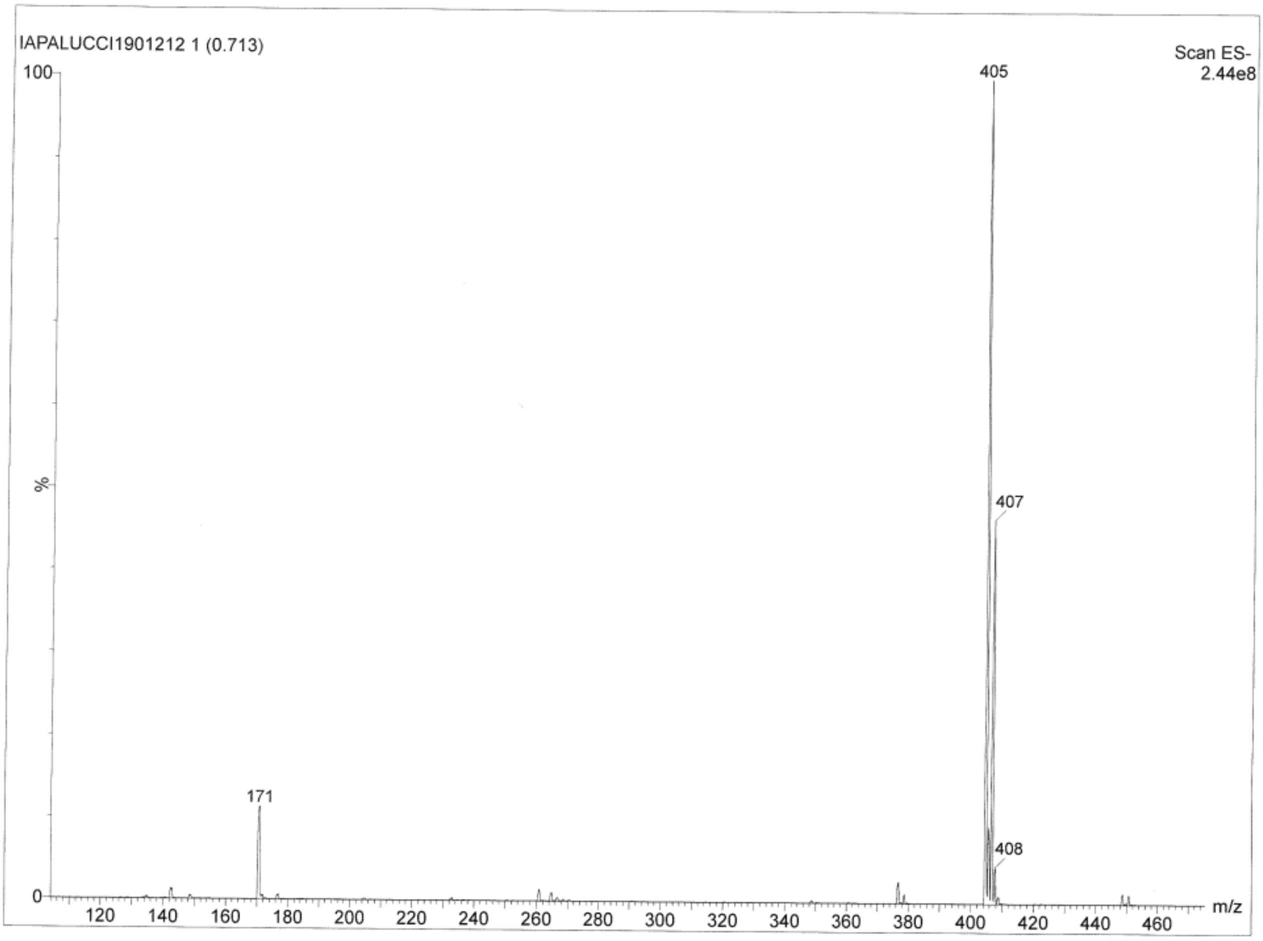

Figure S13. ESI-MS spectrum in CH_3OH (ES–) of [PPN][**2**].

Table S1. Peak assignment of the ESI-MS spectrum (ES–) of [PPN][**2**].

m/z	**Relative intensity**	**Ion**	**Code**
405	100	$[Cu\{Co(CO)_4\}_2]^-$	M
171	5	$[Co(CO)_4]^-$	-

NOTE: The spectrum indicates that in solution is present only the monomer $[Cu\{Co(CO)_4\}_2]^-$ (**2**).

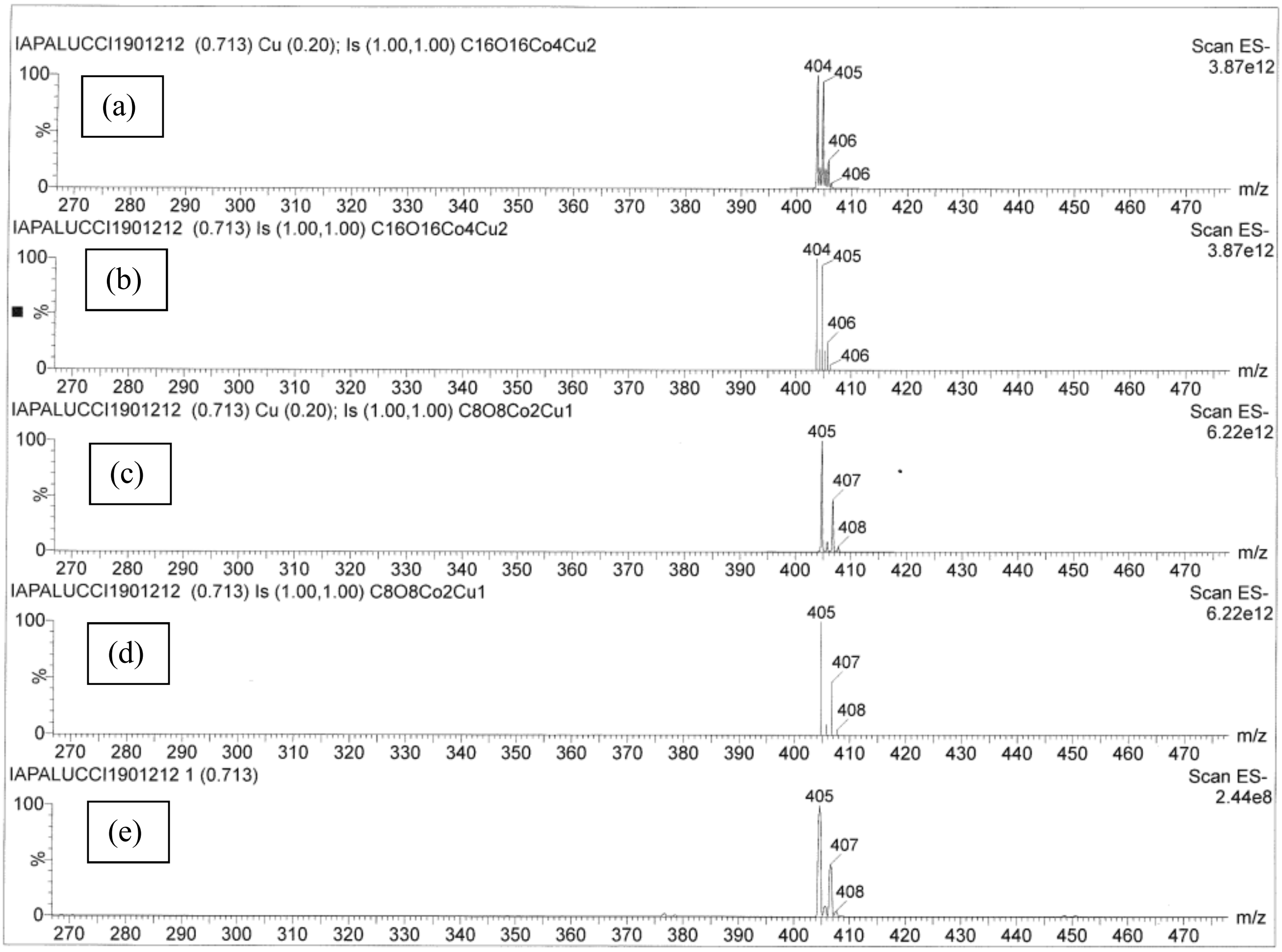

Figure S14. Isotopic pattern of the peak at m/z 405 of the ESI-MS spectrum in CH_3OH (ES–) of [PPN][**2**]. Upper traces (a,b): calculated isotopic patter for $[Cu_2\{Co(CO)_4\}_4]^{2-}$. Middle traces (c,d): calculated isotopic patter for $[Cu\{Co(CO)_4\}_2]^{-}$. Lower trace (e): experimental isotopic pattern.

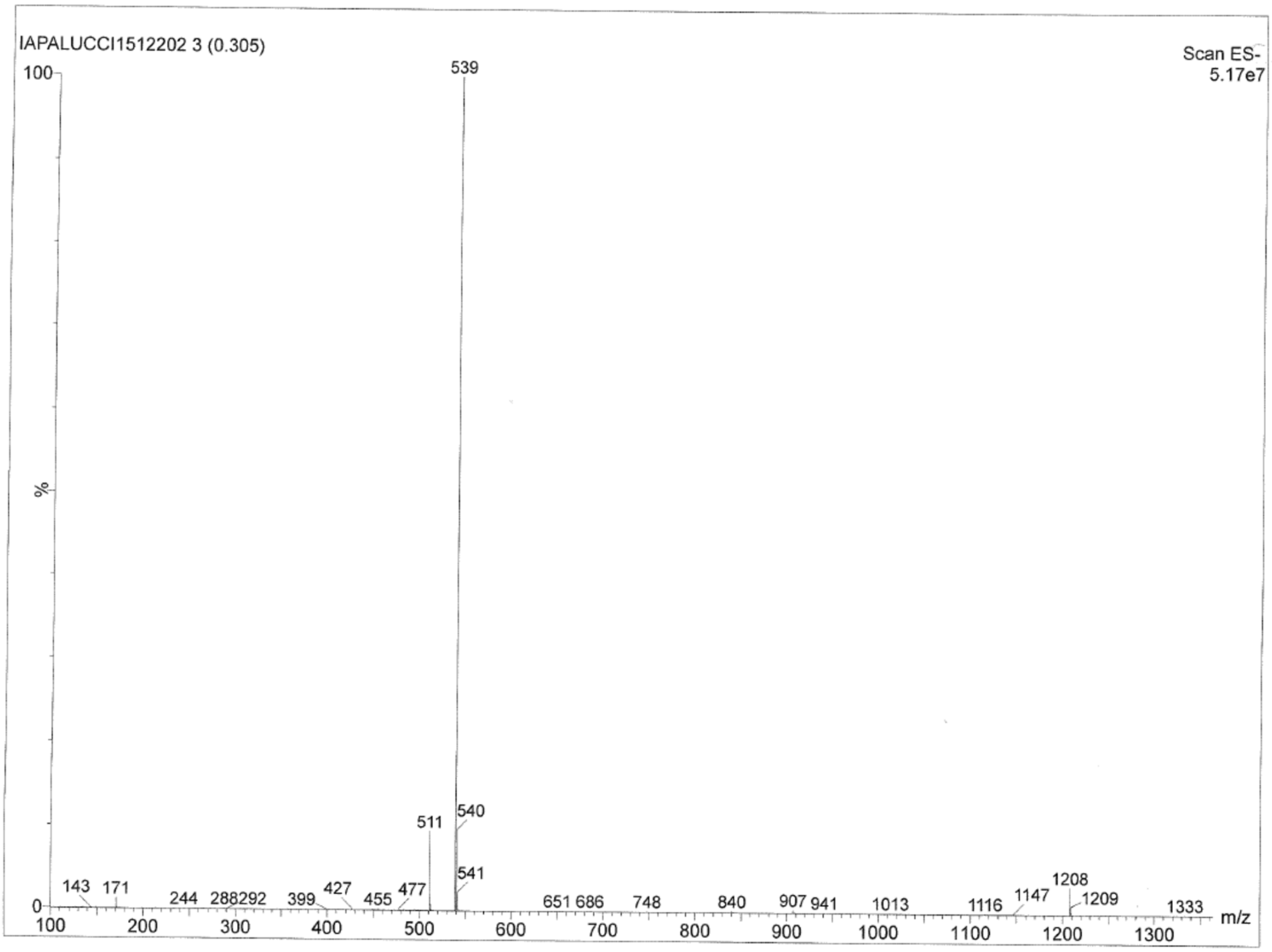

Figure S15. ESI-MS spectrum in CH_3OH (ES–) of [PPN][**4**].

Table S2. Peak assignment of the ESI-MS spectrum (ES–) of [PPN][**4**].

m/z	Relative intensity	Ion	Code
539	100	$[Au\{Co(CO)_4\}_2]^-$	M
511	10	$[Au\{Co(CO)_4\}\{Co(CO)_3\}]^-$	M-CO

NOTE: The spectrum indicates that in solution is present only the monomer $[Au\{Co(CO)_4\}_2]^-$ (**4**).

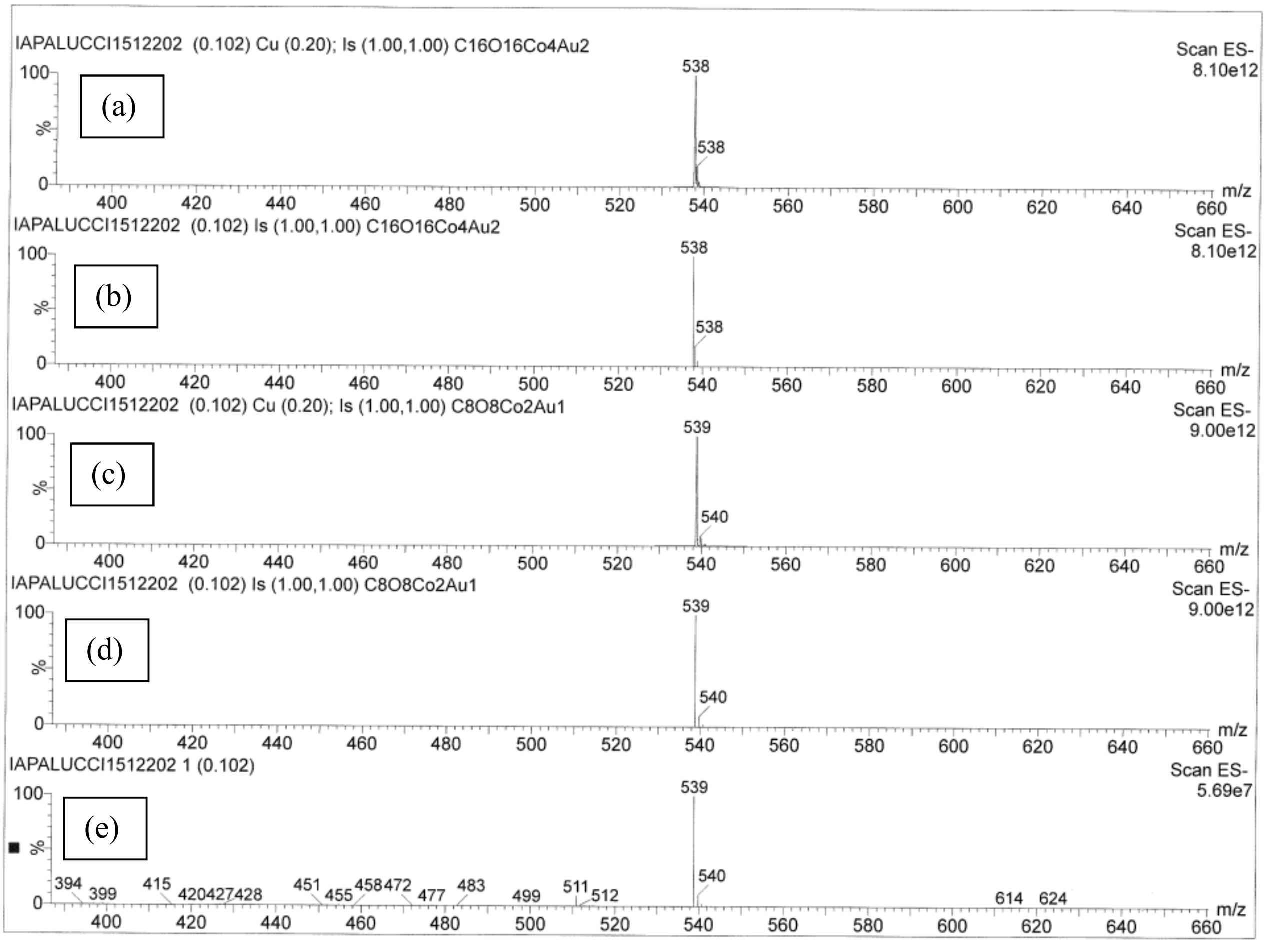

Figure S16. Isotopic pattern of the peak at m/z 539 of the ESI-MS spectrum in CH_3OH (ES–) of [PPN][**4**]. Upper traces (a,b): calculated isotopic patter for $[Au_2\{Co(CO)_4\}_4]^{2-}$. Middle traces (c,d): calculated isotopic patter for $[Au\{Co(CO)_4\}_2]^{-}$. Lower trace (e): experimental isotopic pattern.

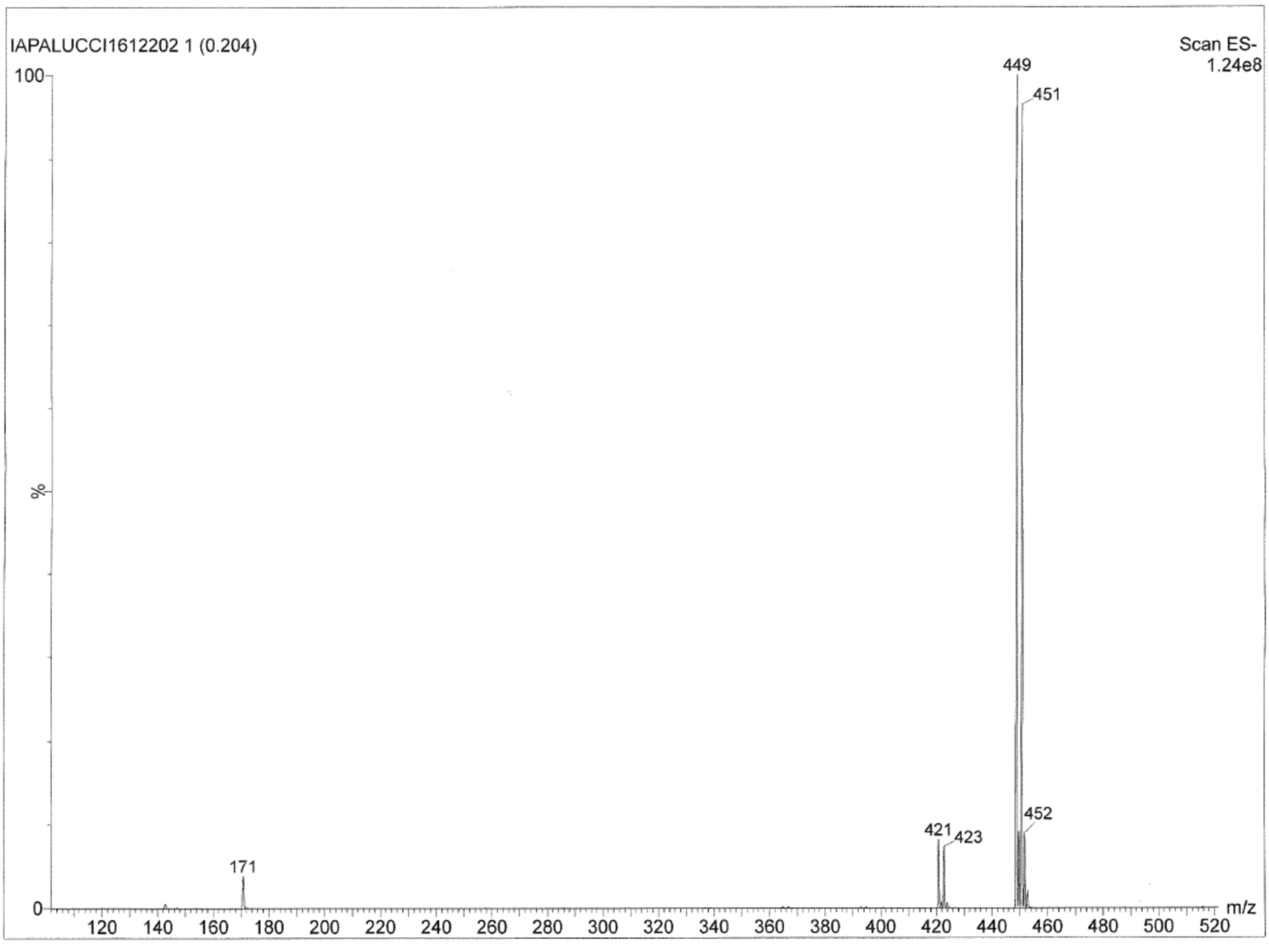

Figure S17. ESI-MS spectrum in CH_3OH (ES–) of $[PPN]_2$[**5**].

Table S3. Peak assignment of the ESI-MS spectrum (ES–) of $[PPN]_2$[**5**].

m/z	Relative intensity	Ion	Code
449	100	$[Ag\{Co(CO)_4\}_2]^-$	M
421	10	$[Ag\{Co(CO)_4\}\{Co(CO)_3\}]^-$	M-CO
171	5	$[Co(CO)_4]^-$	-

NOTE: The spectrum indicates that in solution is present only the monomer $[Ag\{Co(CO)_4\}_2]^-$ (**3**), and not the dimer $[Ag_2\{Co(CO)_4\}_4]^{2-}$ (**5**).

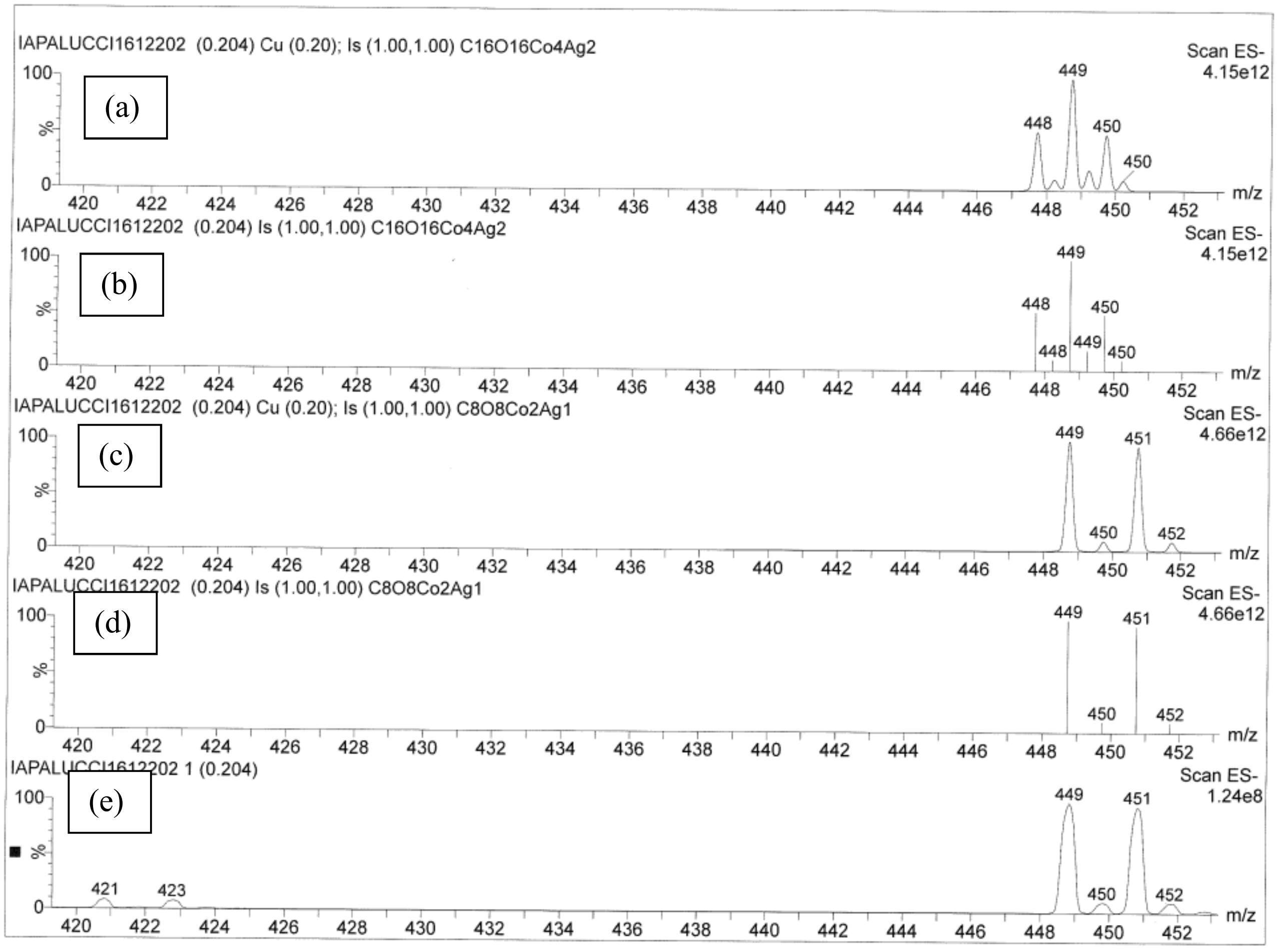

Figure S18. Isotopic pattern of the peak at m/z 449 of the ESI-MS spectrum in CH_3OH (ES–) of [PPN]$_2$[**5**]. Upper traces (a,b): calculated isotopic patter for $[Ag_2\{Co(CO)_4\}_4]^{2-}$. Middle traces (c,d): calculated isotopic patter for $[Ag\{Co(CO)_4\}_2]^{-}$. Lower trace (e): experimental isotopic pattern.

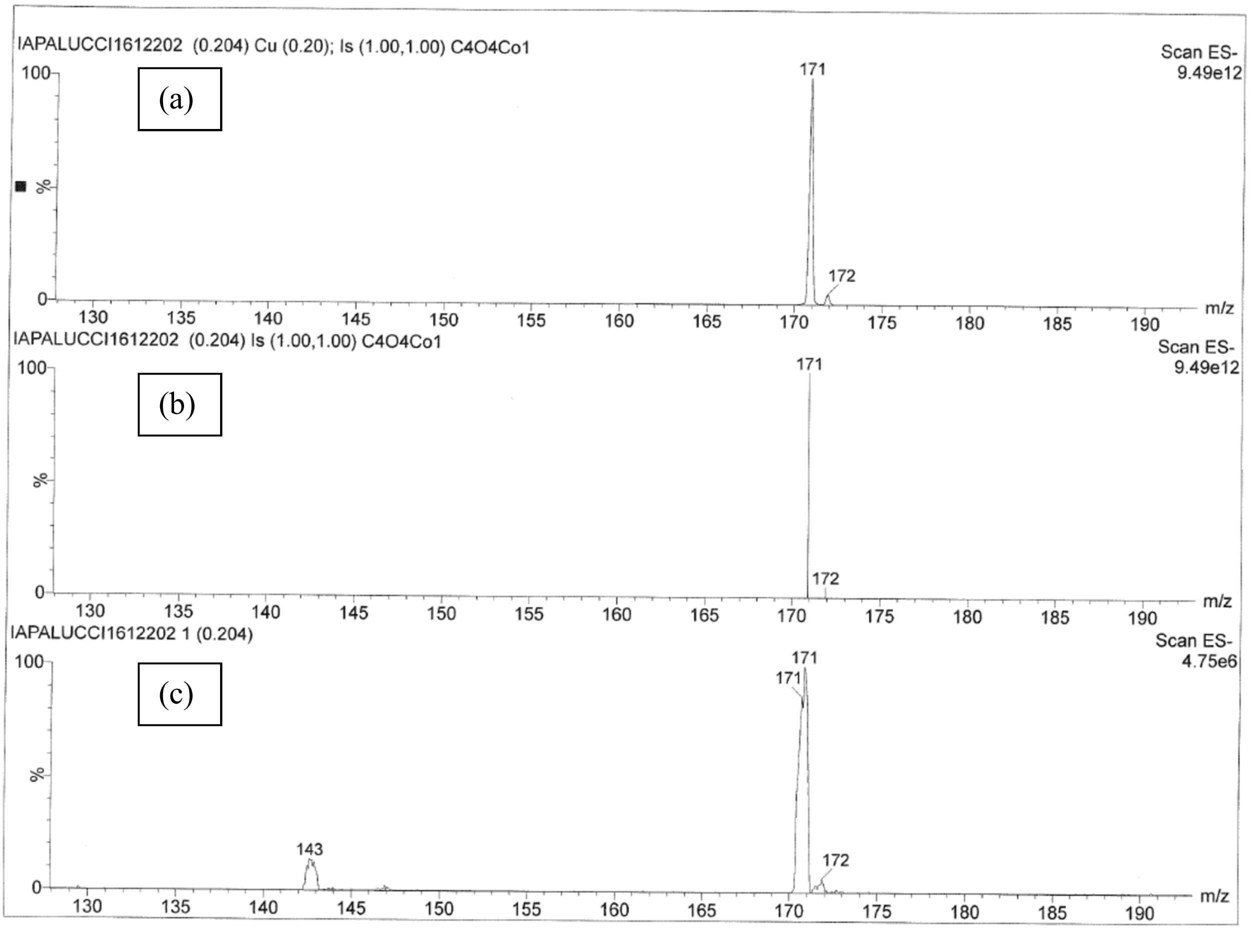

Figure S19. Isotopic pattern of the peak at m/z 171 of the ESI-MS spectrum in CH_3OH (ES–) of $[PPN]_2$[**5**]. Upper traces (a,b): calculated isotopic patter for $[Co(CO)_4]^-$. Lower trace (c): experimental isotopic pattern.

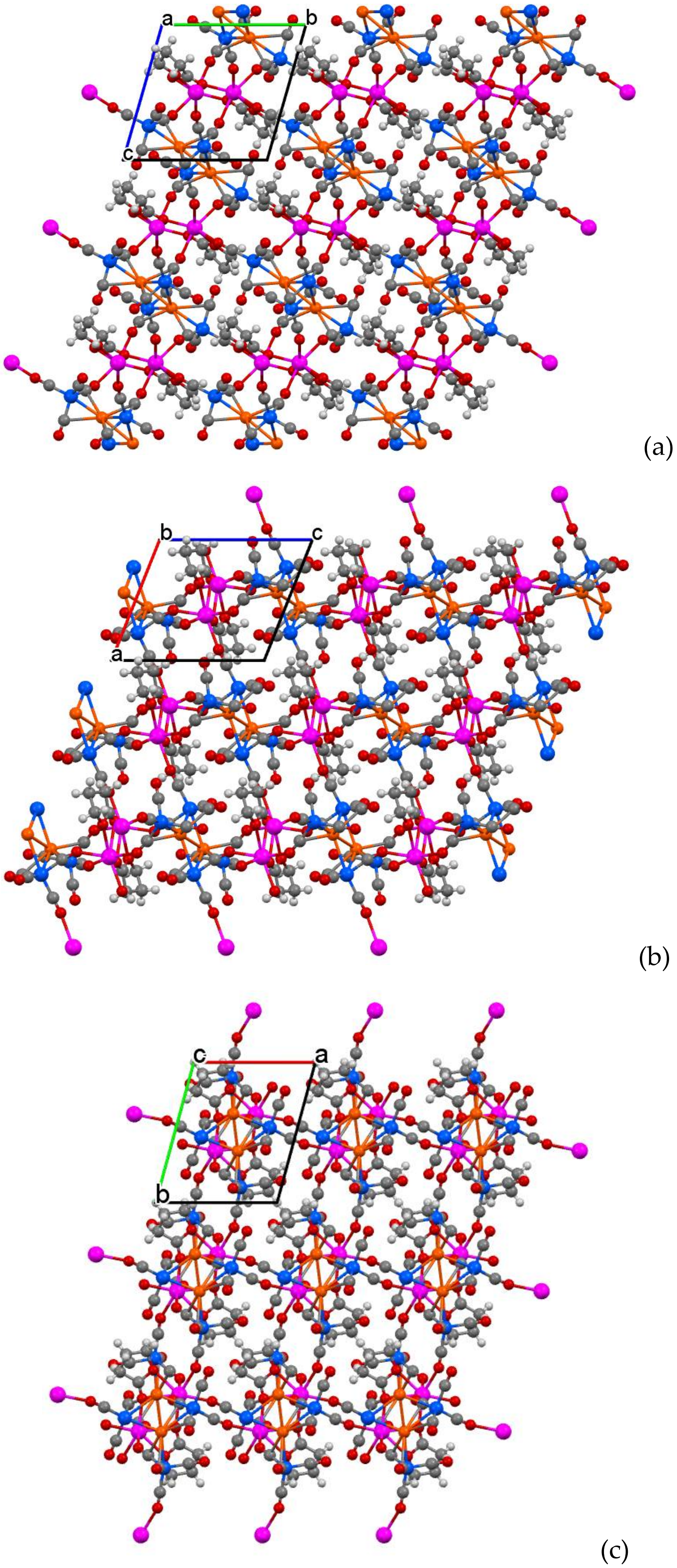

a
b
c
(a)
b
c
a
(b)
c
a
b
(c)

Figure S20. Views of the crystal packing of Na_2[**5**]·$C_4H_6O_2$ (3×3 unit cell) along the crystallographic a, b and c axes (orange, Ag; blue, Co; red, O; grey, C; white, H; purple, Na).

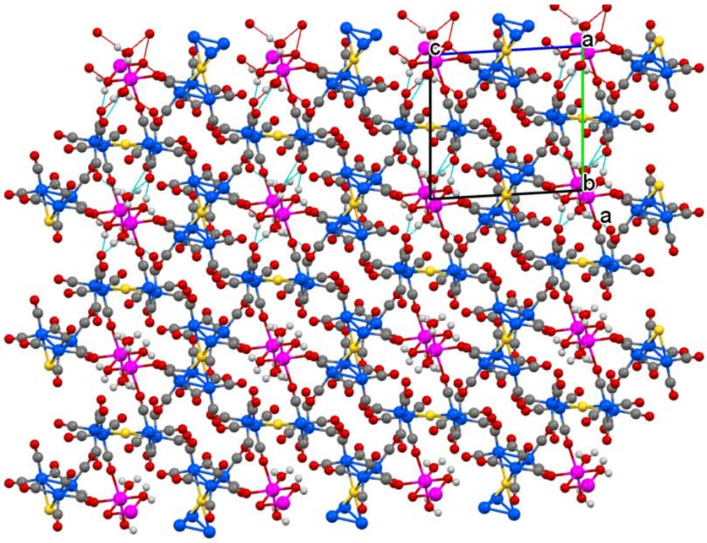

(a)

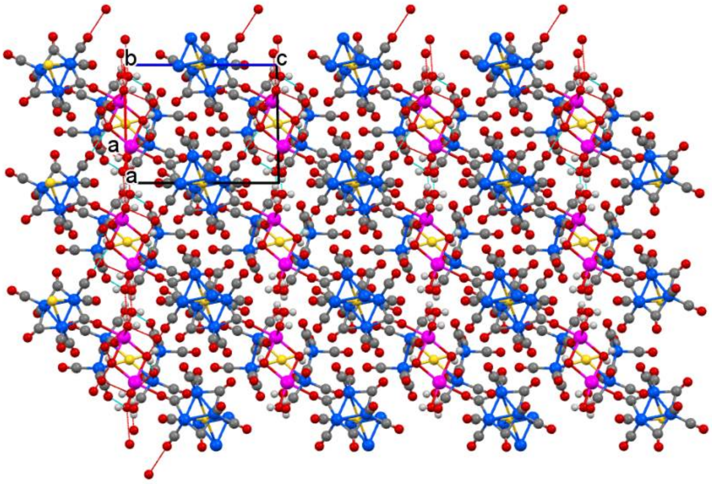

(b)

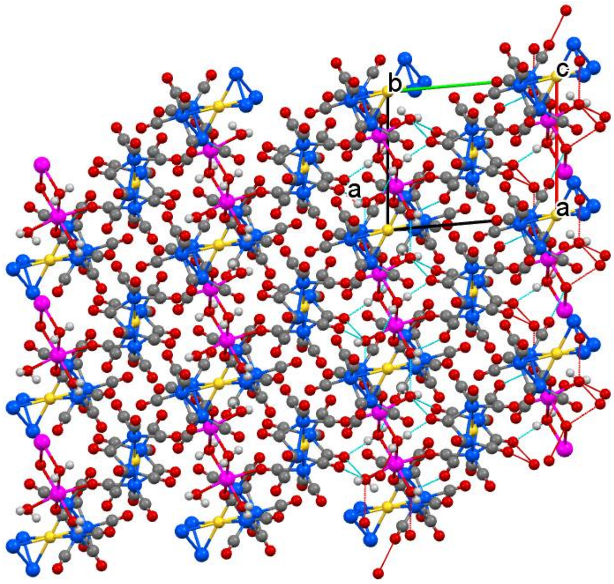

(c)

Figure S21. Views of the crystal packing of Na_2[**7**][**6**]·$6H_2O$ (3×3 unit cell) along the crystallographic a, b and c axes (yellow, Au; blue, Co; red, O; grey, C; white, H; purple, Na). H-bonds are represented as dashed lines.

Table S4. Hydrogen bonds (Å and °) for Na_2[**7**][**6**]·$6H_2O$.

**D-H···A**	**d(D-H)**	**d(H···A)**	**d(D···A)**	**<(DHA)**
O(101)-H(12)···O(15)#1	0.859(10)	2.23(3)	2.992(6)	147(4)
O(102)-H(21)···O(101)#2	0.862(10)	2.46(2)	2.977(6)	120(2)
O(102)-H(22)···O(2)#3	0.866(10)	2.56(5)	3.195(6)	130(5)
O(102)-H(22)···O(14)#4	0.866(10)	2.31(4)	3.033(6)	141(6)
O(103)-H(31)···O(17)#4	0.866(10)	2.006(12)	2.863(5)	170(4)
O(103)-H(32)···O(4)#2	0.868(10)	2.19(3)	2.977(6)	151/(5)

Symmetry transformations used to generate equivalent atoms: #1 -x+1,-y+2,-z; #2 -x+2,-y+2,-z; #3 x,y,z-1; #4 -x+1,-y+1,-z.

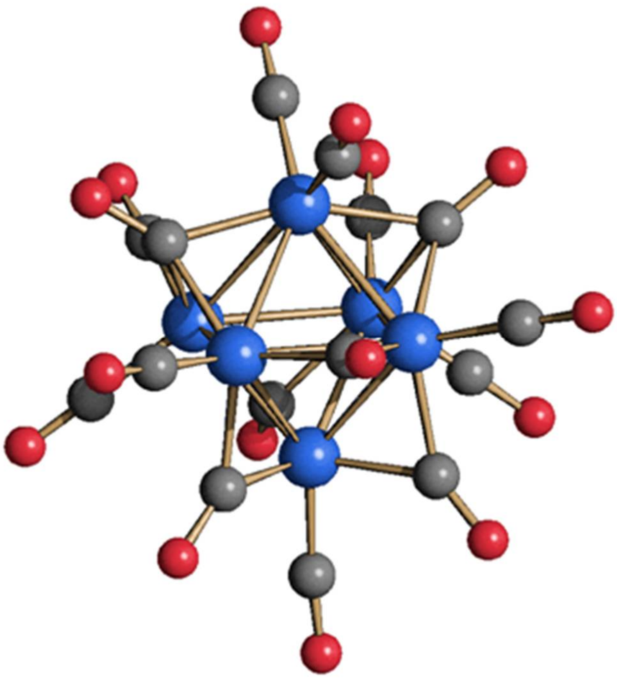

Figure S22. Two views of the molecular structure of the $[Co_6(CO)_{15}]^{2-}$ (**8**) as found in $[NMe_3(CH_2Ph)]_2$[**8**] (blue, Co; red, O; grey, C).

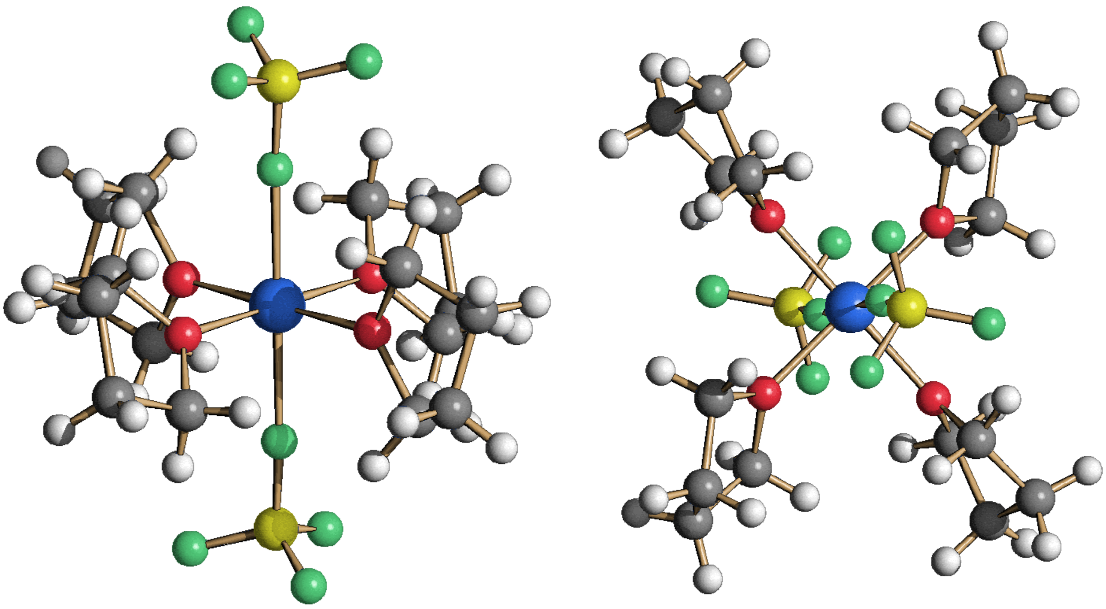

Figure S23. Two views of the molecular structure of the $[Co(THF)_4(BF_4)_2]$ (**9**) complex as found in $[PPN]_2[Co(THF)_4(BF_4)_2][BF_4]_2{\cdot}4CH_2Cl_2$ (yellow, B; blue, Co; green, F; red, O; grey, C; white, H).

X-ray Crystallographic Study.

Crystal data and collection details for [NEt_4][**2**], [NEt_4][**3**], [NEt_4][**4**], Na_2[**5**]·$C_4H_6O_2$, [PPN]$_2$[**5**]·C_5H_{12}, [NBu_4]$_2$[**5**], [NMe_4]$_2$[**5**], Na_2[**7**][**6**}]·6H_2O, [$NMe_3(CH_2Ph)$]$_2$[**8**] and [PPN]$_2$[**9**][BF_4]$_2$·4CH_2Cl_2 are reported in Table S5. The diffraction experiments were carried out on a Bruker APEX II diffractometer equipped with a PHOTON2 detector using Mo–Kα radiation. Data were corrected for Lorentz polarization and absorption effects (empirical absorption correction SADABS). Structures were solved by direct methods and refined by full-matrix least-squares based on all data using F^2. Hydrogen atoms were fixed at calculated positions and refined by a riding model. All non-hydrogen atoms were refined with anisotropic displacement parameters.

[NEt_4][2]: The asymmetric unit of the unit cell contains two halves of two [Cu{Co(CO)$_4$}$_2$]$^-$ anions (located on inversion centers) and one [NEt_4]$^+$ cation (located on a general position). The Cu atoms are disordered over four equally populated (occupancy factor 0.25) positions located on inversion centers. The [NEt_4]$^+$ cation and some CO ligands are disordered and, therefore, they have been split into two positions and refined anysotropically using one occupancy factor per disordered group. Similar *U* parameter restraints have been applied to the [NEt_4]$^+$ cation and CO ligands (SIMU line in SHELXL, s.u. 0.01). Restraints to bond distances were applied as follow (s.u. 0.02): 1.47 Å for C–N and 1.53 Å for C–C in [NEt_4]$^+$.

[NEt_4][3]: The asymmetric unit of the unit cell contains two halves of two [Ag{Co(CO)$_4$}$_2$]$^-$ anions (located on inversion centers) and one [NEt_4]$^+$ cation (located on a general position). The Ag atoms are disordered over four equally populated (occupancy factor 0.25) positions located on inversion centers. The [NEt_4]$^+$ cation is disordered and, therefore, it has been split into two positions and refined anysotropically using one occupancy factor per disordered group. Similar *U* parameter restraints have been applied to the [NEt_4]$^+$ cation and CO ligands (SIMU line in SHELXL, s.u. 0.01). All O atoms have been restrained to isotropic like behaviour (ISOR line in SHELXL, s.u. 0.01). Restraints to bond distances were applied as follow (s.u. 0.02): 1.47 Å for C–N and 1.53 Å for C–C in [NEt_4]$^+$.

[NEt_4][4]: The asymmetric unit of the unit cell contains two halves of two [Au{Co(CO)$_4$}$_2$]$^-$ anions (located on inversion centers) and one [NEt_4]$^+$ cation (located on a general position).

The crystals appeared to be non-merohedrally twinned with a multiple twinning. The TwinRotMat routine of PLATON was used to determine the twinning matrices and to write the reflection data file (.hkl) containing the four twin components. Refinement was performed using the instruction HKLF 5 in SHELXL and three BASF parameters. One Au atom is disordered and, therefore, it has been split into two positions and refined anysotropically using one occupancy factor per disordered group. Similar *U* parameter restraints have been applied to the $[NEt_4]^+$ cation and CO ligands (SIMU line in SHELXL, s.u. 0.01). Restraints to bond distances were applied as follow (s.u. 0.02): 1.47 Å for C–N and 1.53 Å for C–C in $[NEt_4]^+$.

Na_2[5]·$C_4H_6O_2$: The asymmetric unit of the unit cell contains half of a $[Ag_2\{Co(CO)_4\}_4]^{2-}$ anion (located on an inversion center), one Na^+ cation and one $C_4H_6O_2$ molecule (located on general positions).

[PPN]$_2$[5]·C_5H_{12}: The unit cell contains half of a $[Ag_2\{Co(CO)_4\}_4]^{2-}$ anion (located on an inversion center), one $[PPN]^+$ cation and one C_5H_{12} molecule (located on general positions). Similar *U* parameter restraints have been applied to the C_5H_{12} molecule (SIMU line in SHELXL, s.u. 0.01). Restraints to bond distances were applied as follow (s.u. 0.02): 1.53 Å for C–C in C_5H_{12}.

$[NBu_4]_2$[5]: The unit cell contains half of a $[Ag_2\{Co(CO)_4\}_4]^{2-}$ anion (located on an inversion center) and one $[NBu_4]^+$ cation (located on a general position).

$[NMe_4]_2$[5]: The unit cell contains two halves of two $[Ag_2\{Co(CO)_4\}_4]^{2-}$ anions (located on inversion centers) and two $[NMe_4]^+$ cations (located on general positions). The crystals appeared to be non-merohedrally twinned with two twin domains. The TwinRotMat routine of PLATON was used to determine the twinning matrices and to write the reflection data file (.hkl) containing the two twin components. Refinement was performed using the instruction HKLF 5 in SHELXL and one BASF parameters. Similar *U* parameter restraints have been applied to all the C, N and O atoms(SIMU line in SHELXL, s.u. 0.01). All C and O atoms have been restrained to isotropic like behaviour (ISOR line in SHELXL, s.u. 0.01).

Na_2[7][6]·6H_2O: The asymmetric unit of the unit cell contains half of a $[Au\{Co_3(CO)_9\}_2]^-$ anion and half of a $[Au\{Co_2(CO)_2\}]^-$ anion (located on inversion centers), one Na^+ cation and three

H_2O molecules (located on general positions). The H-atoms of the H_2O molecules have been located in the Fourier difference map and refined isotropically using the 1.5-fold U_{iso} value of the parent O-atoms. The O-H distances have been restrained to 0.87 Å and the H···H contacts to 1.4 Å(s.u. 0.02). The location of the H-atoms is corroborated by the presence of H-bonds involving other H_2O molecules and some CO ligands.

[NMe$_3$(CH$_2$Ph)]$_2$[8]: The asymmetric unit of the unit cell contains one $[Co_6(CO)_{15}]^{2-}$ anion and two $[NMe_3(CH_2Ph)]^+$ cations located on general positions.

[PPN]$_2$[9][BF$_4$]$_2$·4CH$_2$Cl$_2$: The asymmetric unit of the unit cell contains half of a $[Co(THF)_4(BF_4)_2]$ molecule (located on an inversion center), one $[PPN]^+$ cation, one $[BF_4]^-$ anion and two CH_2Cl_2 molecules (all located on general positions).

Table S5

Crystal data and experimental details for [NEt$_4$][**2**], [NEt$_4$][**3**], [NEt$_4$][**4**], Na$_2$[**5**]·$C_4H_6O_2$, [PPN]$_2$[**5**]·C_5H_{12}, [NBu$_4$]$_2$[**5**], [NMe$_4$]$_2$[**5**], Na$_2$[**7**][**6**]·6H_2O, [NMe$_3$(CH$_2$Ph)]$_2$[**8**] and [PPN]$_2$[**9**][BF$_4$]$_2$·4CH$_2$Cl$_2$

	**[NEt$_4$][2]**	**[NEt$_4$][3]**	**[NEt$_4$][4]**
Formula	$C_{16}H_{20}Co_2CuNO_8$	$C_{16}H_{20}AgCo_2NO_8$	$C_{16}H_{20}AuCo_2NO_8$
Fw	535.73	580.06	669.16
T, K	100(2)	100(2)	100(2)
λ, Å	0.71073	0.71073	0.71073
Crystal system	Triclinic	Triclinic	Triclinic
Space Group	$P\bar{1}$	$P\bar{1}$	$P\bar{1}$
a, Å	8.0726(18)	8.1192(12)	8.1440(18)
b, Å	8.1234(19)	8.1305(12)	8.1511(17)
c, Å	17.566(4)	17.461(3)	17.439(4)
, °	103.155(11)	102.529(9)	103.459(14)
β, °	103.059(11)	102.497(9)	103.358(10)
, °	90.743(12)	93.750(6)	90.126(11)
Cell Volume, Å^3	1090.2(4)	1090.9(3)	1093.5(4)
Z	2	2	2
D_c, g cm^{-3}	1.632	1.766	2.032

μ, mm^{-1}	2.509	2.430	8.230
F(000)	540	576	640
Crystal size, mm	0.22×0.16×0.13	0.21×0.16×0.12	0.18×0.12×0.10
θ limits, °	2.450–24.996	2.460–25.093	2.473–25.026
Reflections collected	11126	12348	9495
Independent reflections	3794 [R_{int} = 0.2068]	3816 [R_{int} = 0.0908]	3761 [R_{int} = 0.1197]
Completeness to θ max	98.6%	98.6%	99.8%
Data / restraints / parameters	3794 / 410 / 357	3816 / 370 / 317	3761 / 171 / 266
Goodness on fit on F^2	1.072	1.118	1.107
R_1 (I > 2σ(I))	0.1559	0.1056	0.1329
wR_2 (all data)	0.4051	0.2666	0.3727
Largest diff. peak and hole, e $Å^{-3}$	1.643 / -1.325	1.723 / –1.138	5.591 / –3.420

	**$Na_2[5]\cdot C_4H_6O_2$**	**$[PPN]_2[5]\cdot C_5H_{12}$**	**$[NBu_4]_2[5]$**	**$[NMe_4]_2[5]$**
Formula	$C_{27}H_{12}Ag_2Co_4Na_2O_{20}$	$C_{93}H_{72}Ag_2Co_4N_2O_{16}P_4$	$C_{48}H_{72}Ag_2Co_4N_2O_{16}$	$C_{24}H_{24}Ag_2Co_4N_2O_{16}$
Fw	1117.78	2048.86	1384.53	1047.91
T, K	100(2)	100(2)	100(2)	100(2)
λ, Å	0.71073	0.71073	0.71073	0.71073
Crystal system	Triclinic	Monoclinic	Monoclinic	Monoclinic
Space Group	$P\bar{1}$	$C2/c$	$P2_1/n$	$P2_1/c$
a, Å	8.9246(3)	25.366(8)	12.6081(4)	12.3399(18)
b, Å	10.3336(4)	14.902(5)	16.6822(5)	13.799(2)
c, Å	10.4920(4)	23.330(8)	13.7182(4)	20.638(3)
, °	101.5370(10)	90	90	90
β, °	108.6820(10)	97.690(14)	94.3590(10)	96.561(5)
, °	100.3630(10)	90	90	90
Cell Volume, $Å^3$	866.53(6)	8739(5)	2877.01(15)	3491.1(9)

Z	1	4	2	4
D_c, g cm^{-3}	2.142	1.557	1.598	1.994
μ, mm^{-1}	3.084	1.322	1.857	3.026
F(000)	540	4136	1408	2048
Crystal size, mm	0.21×0.18×0.15	0.19×0.18×0.16	0.24×0.21×0.18	0.23×0.16×0.12
θ limits, °	2.085–27.000	1.589–25.999	2.115–27.994	1.661–25.093
Reflections collected	18175	55909	63321	59252
Independent reflections	3786 [R_{int} = 0.0328]	8595 [R_{int} = 0.0343]	6946 [R_{int} = 0.0438]	6221 [R_{int} = 0.1776]
Completeness to θ max	99.9%	100.0%	99.9%	99.9%
Data / restraints / parameters	3786 / 0 / 235	8595 / 37 / 568	6946 / 0 / 329	6221 / 390 / 442
Goodness on fit on F^2	1.078	1.087	1.087	1.113
R_1 (I > 2σ(I))	0.0169	0.0283	0.0165	0.1402
wR_2 (all data)	0.0389	0.0658	0.0397	0.3674
Largest diff. peak and hole, e Å^{-3}	0.937 / -0.575	1.040 / -0.391	0.381 / -0.400	5.228 / -4.613

	**Na_2[7][6]·6H_2O**	**[$NMe_3(CH_2Ph)]_2$[8]**	**[PPN]$_2$[9][BF_4]$_2$·4CH_2Cl_2**
Formula	$C_{32}H_{12}Au_2Co_{10}Na_2O_{38}$	$C_{35}H_{32}Co_6N_2O_{15}$	$C_{92}H_{100}B_4Cl_8CoF_{16}N_2O_4P_4$
Fw	2033.63	1074.20	2111.38
T, K	100(2)	100(2)	100(2)
λ, Å	0.71073	0.71073	0.71073
Crystal system	Triclinic	Monoclinic	Triclinic
Space Group	$P\bar{1}$	$P2_1/n$	$P\bar{1}$
a, Å	9.4702(4)	12.3218(6)	11.5987(9)
b, Å	11.5884(4)	14.0851(6)	14.6213(11)
c, Å	12.2012(5)	23.0096(10)	14.9774(11)
, °	86.5840(10)	90	104.502(3)
β, °	88.7660(10)	101.963(2)	97.931(3)
, °	84.8610(10)	90	93.244(3)
Cell Volume, Å^3	1331.09(9)	3906.7(3)	2424.5(3)

Z	1	4	1
D_c, g cm^{-3}	2.537	1.826	1.446
μ, mm^{-1}	8.635	2.561	0.545
F(000)	958	2152	1085
Crystal size, mm	0.14×0.13×0.11	0.15×0.13×0.08	0.22×0.19×0.16
θ limits, °	1.672–25.099	1.705–26.000	1.445–25.998
Reflections collected	23318	74489	32263
Independent reflections	4685 [R_{int} = 0.1381]	7662 [R_{int} = 0.0824]	9506 [R_{int} = 0.0549]
Completeness to θ max	98.7%	100.0%	99.7%
Data / restraints / parameters	4685 / 35 / 400	7662 / 0 / 529	9506 / 0 / 593
Goodness on fit on F^2	0.932	1.096	1.092
R_1 ($I > 2\sigma(I)$)	0.0256	0.0269	0.0995
wR_2 (all data)	0.0565	0.0555	0.2727
Largest diff. peak and hole, e Å^{-3}	1.153 / -1.636	0.568 / -0.301	2.429 / -2.346

DFT optimized geometries (cartesian coordinates, in Å)

2, $[Cu\{Co(CO)_4\}_2]^-$

Cu -0.0000060000 -0.0001660000 0.0003760000
Co 2.3850700000 -0.0000230000 -0.0001650000
Co -2.3850540000 0.0000690000 0.0000250000
C 4.1491480000 0.0002850000 0.0012190000
C 1.9457960000 1.5496340000 0.7626690000
C 1.9459920000 -1.4361060000 0.9595970000
C 1.9475190000 -0.1138280000 -1.7240810000
C -4.1491470000 -0.0002810000 -0.0007600000
C -1.9470310000 0.1148620000 1.7237470000
C -1.9464820000 1.4356060000 -0.9607990000
C -1.9458640000 -1.5499460000 -0.7621920000
O 5.3084880000 0.0005940000 0.0021460000
O 1.7545850000 2.5769820000 1.2684040000
O 1.7547250000 -2.3881440000 1.5958560000
O 1.7568200000 -0.1892450000 -2.8667420000
O -5.3084820000 -0.0004890000 -0.0013090000
O -1.7561940000 0.1908410000 2.8663600000
O -1.7553080000 2.3872390000 -1.5976710000
O -1.7546160000 -2.5775000000 -1.2674800000

3, $[Ag\{Co(CO)_4\}_2]^-$

Ag 0.0000240000 0.0001680000 0.0000930000
Co 2.6101330000 0.0000150000 0.0000040000
Co -2.6101360000 -0.0000300000 -0.0000080000
C 4.3721340000 -0.0003490000 -0.0001990000
C 2.2204570000 -1.6856190000 -0.4405890000
C 2.2208900000 1.2245580000 -1.2394790000
C 2.2210720000 0.4613220000 1.6802240000
C -4.3721440000 0.0003170000 0.0003340000
C -2.2210570000 -0.4616210000 -1.6801480000
C -2.2208120000 -1.2246630000 1.2393580000
C -2.2206110000 1.6857060000 0.4402910000
O 5.5312230000 -0.0005870000 -0.0003120000
O 2.0781660000 -2.7999140000 -0.7318470000
O 2.0788250000 2.0339960000 -2.0588420000
O 2.0791230000 0.7662380000 2.7909010000
O -5.5312320000 0.0005610000 0.0005530000

O -2.0790370000 -0.7667760000 -2.7907470000
O -2.0787130000 -2.0342590000 2.0585570000
O -2.0784310000 2.8000670000 0.7313600000

4, $[Au\{Co(CO)_4\}_2]^-$

Au 0.0000740000 0.0004140000 0.0006670000
Co 2.5918340000 -0.0001500000 -0.0001450000
Co -2.5916930000 -0.0005400000 0.0005970000
C 4.3529810000 -0.0021070000 -0.0000570000
C 2.2212970000 -1.4291140000 -1.0148930000
C 2.2235070000 1.5935310000 -0.7305630000
C 2.2221390000 -0.1631520000 1.7449770000
C -4.3529040000 -0.0015580000 0.0022670000
C -2.2231950000 0.1617190000 -1.7447450000
C -2.2215480000 -1.5937190000 0.7314920000
C -2.2224050000 1.4319310000 1.0108160000
O 5.5111680000 -0.0035560000 0.0000610000
O 2.0836210000 -2.3668220000 -1.6809750000
O 2.0867090000 2.6392640000 -1.2097510000
O 2.0849510000 -0.2701930000 2.8902550000
O -5.5110750000 -0.0024450000 0.0032210000
O -2.0869500000 0.2680670000 -2.8901760000
O -2.0841600000 -2.6393940000 1.2106210000
O -2.0853700000 2.3751670000 1.6691620000

5, $[Ag_2\{Co(CO)_4\}_4]^{2-}$

Ag -1.5657420000 -0.0114980000 0.0680410000
Co 0.0036740000 -2.4309830000 0.3239230000
Co -4.2825820000 -0.0055580000 -0.3651760000
C -0.1188650000 -1.9378450000 -1.3933240000
C -1.0872270000 -3.8304360000 0.3463140000
C 1.6270840000 -3.1262510000 0.5414680000
C -0.3884310000 -1.7069370000 1.9123430000
C -3.9320240000 -1.6757030000 -0.8830070000
C -4.0355830000 0.3484270000 1.3633090000
C -6.0280540000 0.1026380000 -0.5150290000
C -3.7106000000 1.2151010000 -1.5308520000
O -0.1844820000 -1.8167180000 -2.5467470000
O -1.7347820000 -4.7898180000 0.3929990000
O 2.6183260000 -3.7028080000 0.7074160000
O -0.6155080000 -1.4462840000 3.0213660000
O -3.8282520000 -2.7682570000 -1.2614940000
O -4.0248050000 0.5863760000 2.5016800000
O -7.1855100000 0.1707740000 -0.6192000000
O -3.4671950000 2.0186440000 -2.3333680000
Ag 1.5657900000 0.0119330000 0.0675020000
Co -0.0036200000 2.4317910000 0.3232850000
Co 4.2825640000 0.0044280000 -0.3651120000
C 0.3933670000 1.7095770000 1.9113750000
C 1.0839970000 3.8337870000 0.3424680000
C -1.6278230000 3.1241540000 0.5442090000
C 0.1178960000 1.9376900000 -1.3937520000
C 3.9338880000 1.6764500000 -0.8781240000
C 3.7089040000 -1.2126910000 -1.5336550000
C 6.0278600000 -0.1055110000 -0.5158070000
C 4.0355890000 -0.3536530000 1.3625420000
O 0.6232420000 1.4503660000 3.0201590000
O 1.7292650000 4.7948180000 0.3869210000
O -2.6194150000 3.6994100000 0.7126370000
O 0.1829350000 1.8159410000 -2.5471550000
O 3.8313890000 2.7702770000 -1.2532750000
O 3.4644970000 -2.0140660000 -2.3380380000
O 7.1851990000 -0.1748030000 -0.6204830000
O 4.0247050000 -0.5944240000 2.5003050000

$[Cu_2\{Co(CO)_4\}_4]^{2-}$

Cu -1.3946300000 -0.0132980000 0.0001500000
Co -0.1172780000 -2.3080160000 0.0000400000
Co -3.9660670000 0.0484020000 0.0002190000
C -0.4367920000 -1.6771710000 -1.6383140000
C -1.1325990000 -3.7624550000 -0.0012840000
C 1.5213440000 -2.9986610000 0.0012070000
C -0.4384280000 -1.6778350000 1.6383250000
C -4.1522580000 -1.7235150000 0.0006710000
C -3.3243710000 0.6232060000 1.5559580000
C -5.5896120000 0.7175640000 -0.0000050000
C -3.3243960000 0.6219280000 -1.5559970000
O -0.6287920000 -1.4711460000 -2.7653970000
O -1.7071450000 -4.7685140000 -0.0022340000
O 2.5073710000 -3.6069840000 0.0020330000
O -0.6311060000 -1.4721730000 2.7653600000
O -4.3614640000 -2.8651670000 0.0009170000
O -3.0739710000 1.0256660000 2.6187330000
O -6.6748350000 1.1401170000 -0.0001340000
O -3.0739240000 1.0234600000 -2.6191060000
Cu 1.3947360000 0.0133750000 -0.0004570000
Co 0.1172360000 2.3080120000 -0.0001100000
Co 3.9660850000 -0.0484240000 0.0001230000
C 0.4372350000 1.6773760000 1.6382380000
C 1.1324370000 3.7625420000 0.0002970000
C -1.5214140000 2.9986030000 -0.0008840000
C 0.4380730000 1.6776780000 -1.6383880000
C 4.1519280000 1.7235290000 0.0001830000
C 3.3245860000 -0.6231980000 -1.5557120000
C 5.5898030000 -0.7171680000 0.0000480000
C 3.3244600000 -0.6225330000 1.5561370000
O 0.6294850000 1.4713560000 2.7652750000
O 1.7068830000 4.7686580000 0.0004880000
O -2.5074440000 3.6069190000 -0.0015270000
O 0.6305520000 1.4720780000 -2.7654730000
O 4.3609080000 2.8652220000 0.0003670000
O 3.0742390000 -1.0256900000 -2.6184890000
O 6.6751280000 -1.1394560000 -0.0000310000
O 3.0738140000 -1.0244580000 2.6190510000
